# An automated high-resolution screening platform identifies regulators of anchor cell invasion in *C. elegans*

**DOI:** 10.1126/sciadv.aef6546

**Published:** 2026-09-18

**Authors:** Simon Berger, Silvan Spiri, Evelyn Lattmann, Stefanie Engleitner, Mitchell P. Levesque, Andrew deMello, Alex Hajnal

**Affiliations:** ^1^ Department of Molecular Life Science, University of Zürich, Winterthurerstrasse 190, 8057 Zürich, Switzerland.; ^2^ Department of Dermatology, University of Zürich, University of Zürich Hospital, Wagistrasse 18, 8952 Schlieren, Switzerland.; ^3^ Institute for Chemical- and Bioengineering, ETH Zürich, Vladimir Prelog Weg 1, 8093 Zürich, Switzerland.

## Abstract

Microfluidic devices are valuable tools for live imaging. However, widespread adoption of microfluidic-based screening methods has been limited by the complexity of the existing techniques. Here, we introduce a user-friendly, high-throughput, and high-resolution automated imaging system for *C. elegans*. We demonstrate the system’s capabilities in an RNA interference (RNAi) screen, combined with neural network–based phenotypic scoring. We evaluated the effects of RNAi targeting 193 candidate genes on anchor cell (AC) invasion, a model for basement membrane (BM) breaching that shares similarities with tumor cell invasion during cancer metastasis. Over 40,000 animals were imaged at subcellular resolution and scored using a custom neural network classifier with an accuracy of over 92%. The screen identified 41 of 52 genes previously known to control AC invasion, along with 51 additional regulators of invasion. This automated imaging and classification system enables researchers to perform forward mutagenesis, RNAi, and drug screens in *C. elegans* with much greater speed and higher resolution than previously possible.

## INTRODUCTION

The nematode *Caenorhabditis elegans* offers many unique advantages as a model organism for biological research. Specifically, its small size, transparency, low maintenance costs, and availability of genome engineering tools, such as RNA interference (RNAi) and CRISPR, make it an ideal organism for various large-scale genetic screening applications. Therefore, *C. elegans* is a compelling animal model for functional screening, serving as an intermediate between cell culture and vertebrate models. However, screening applications have been limited to lifespan, behavior, or clear morphological readouts, usually assessed at low magnification at the population level. Large-scale image-based screening at cellular or subcellular resolution has remained challenging due to a lack of suitable sample mounting methods. Most *C. elegans* imaging has been conducted manually using agar pads, which are simple to produce but tedious and time-consuming to use for imaging.

Two microfluidic, image-based screening strategies have been developed to address these limitations. Chung *et al.* (*1*), later refined by San-Miguel *et al.* (*2*), introduced worms from a reservoir into a single imaging channel, immobilized them using hydraulic valves and cooling, and optionally sorted them based on phenotype. This setup enables imaging of several hundred animals per hour and the recovery of specific individuals. However, its complex hardware requirements and single-channel design make it prone to clogging and contamination. Mondal *et al.* (*3*) instead trapped thousands of worms in a 96-well microfluidic array, each with 40 tapered trap channels, enabling imaging of up to 5000 animals per hour at low magnification. However, the need for constant pressure during imaging results in variable worm positioning in the channels, restricting it to low-magnification optics (≤10×) and requiring a custom hardware setup. Overall, both systems substantially increase throughput compared to agar-pad imaging but remain technically demanding, limiting their widespread adoption.

To overcome the limitations of the existing methods, we integrated the simple-to-use microfluidic imaging devices described in Spiri *et al.* (*4*) into a fully automated, high-speed image-acquisition system, combined with neural network–based image classification. This system is well-suited for large-scale experimentation, such as RNAi and drug compound screens. Moreover, individual animals of interest can be labeled inside the devices and recovered for further propagation, making it suitable for forward genetic screening.

To demonstrate the capabilities of our method, we conducted an RNAi screen to investigate the effects of selected candidate genes on basement membrane (BM) breaching during anchor cell (AC) invasion. The AC is a specialized organizer cell in the somatic gonad of the hermaphrodite larva. After inducing the vulval precursor cells (VPCs) during the L2 stage, the AC breaches the two BMs separating the somatic gonad from the underlying VPCs during the L3 stage (*5*). AC invasion involves the precise alignment of the AC relative to the 1° fated P6.p descendants, the rearrangement of the actomyosin cytoskeleton to form an invadopodium (*6*), and the secretion of metalloproteinases (*7*, *8*) and other factors destabilizing the BM underneath the AC. This results in the spatially and temporally reproducible breaching of the BMs, which allows the AC to establish direct contact with the differentiating 1° vulval cells during vulval morphogenesis (*9*). AC invasion in *C. elegans* exhibits similarities to the epithelial-to-mesenchymal transition cells undergo during normal development and to the invasion of tumor cells during metastasis formation (*10*, *11*). Many genes controlling AC invasion, such as the AP-1 transcription factor FOS-1 and the EVI1 proto-oncogene *egl-43*, encode homologs of human genes associated with tumor cell invasion. Using neural network–based phenotypic scoring of more than 40,000 RNAi-treated animals, we identified 41 of 52 genes previously known to control AC invasion, as well as 51 previously unknown invasion regulators.

## RESULTS

### Design of microfluidic devices for automated imaging

**Fig. 1.**
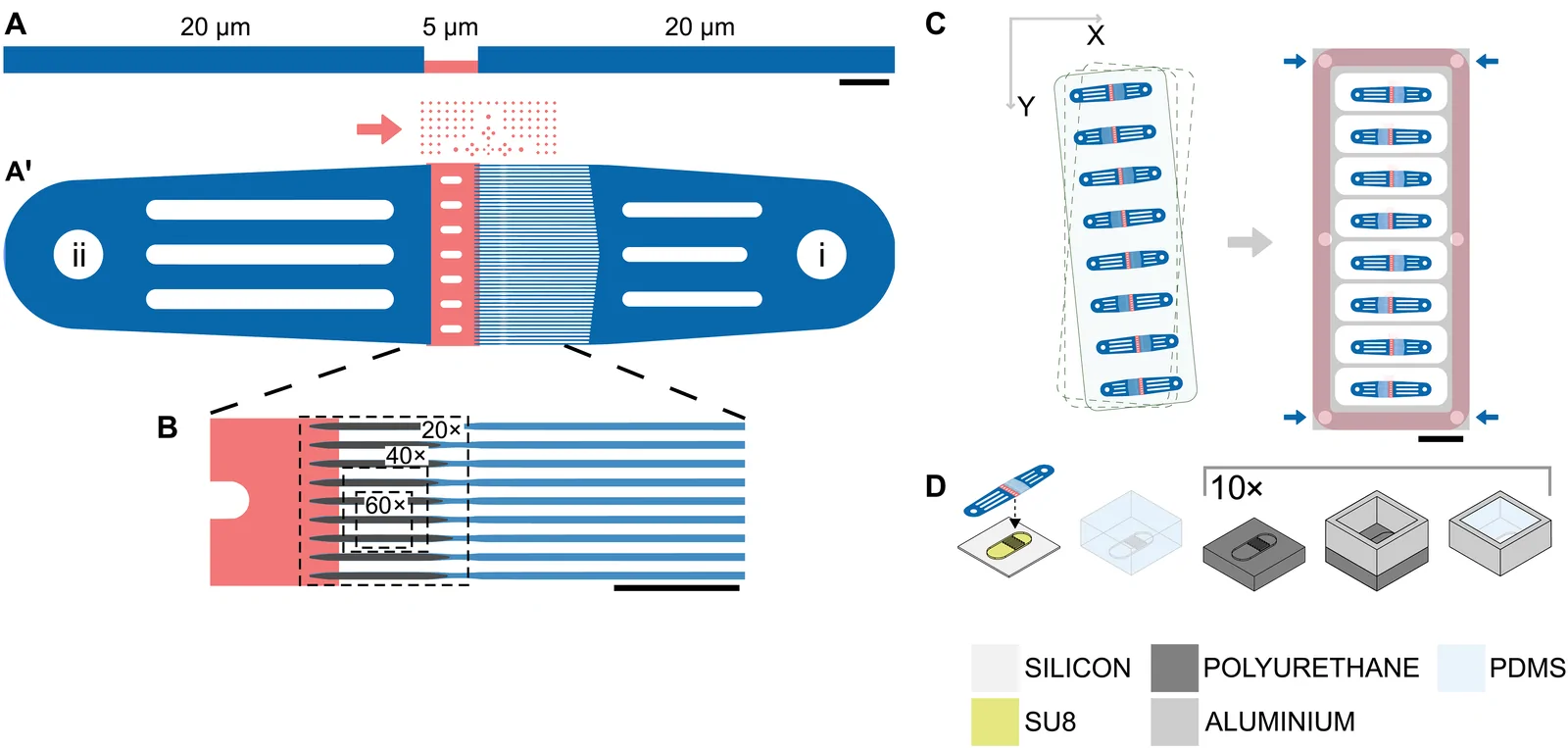
Microfluidic device design and fabrication. (**A** and **A’**) Device overview. (A) Side view of the trap array. (A’) Top view of the trap array. Worms are loaded manually from (i) and pushed into a parallel channel array, which houses up to 49 individual animals, with liquid exiting the device through the outlet (ii). Alignment and position markers are placed alongside each functional unit (arrowhead). (**B**) Magnified view of the parallel trap channel array and imaging region. All trap channels (blue) connect to the same lower height channel (red). Individual worms (gray) are held in the parallel trap channel array with dimensions carefully chosen to match the animals’ size and shape at the desired stage. The overlay shows the ROI at different magnifications (20×, 40×, and 60×). (**C**) Device layout, featuring eight functional units for a total of 392 animals per device. (C and D) Traditional PDMS devices display large variations in overall outer dimensions and tend to tilt when placed on a microscope. Devices fabricated using the presented modified scheme exhibit minimal variation and tilt, and are consistently mounted on the microscope using the aluminum support frame. (**D**) Modified device fabrication process. From left to right, starting with a silicon/SU8 master mold (overlayed with a single device unit relative to the schematic), a PDMS mold is created. Multiple identical molds are then produced from the PDMS mold. Final devices are assembled by precisely aligning the aluminum frame to the master mold and filling it with PDMS. Colors indicate the used materials as indicated. Scale bars: (A) 1000, (B) 500, and (C) 5000 μm.

The microfluidic imaging device consists of an array of 49 parallel trap channels positioned between a single inlet and outlet (trap array) (Fig. 1, A and A’). Immobilization is achieved passively through a combination of channel height and width (H/W ratio 0.7 to 0.8), with the channels closely matching the size and shape of the trapped animal, ensuring minimal motion and proper lateral orientation. All worms are trapped at the end of each trap channel in a well-defined imaging region by a sharp reduction in channel height (Fig. 1, A and A’, red). The regular arrangement of the trap channels, tight spacing of the channels and parallel worm orientation allow simultaneous imaging of multiple animals in one field of view (FOV) (Fig. 1B). Throughput is further increased with eight identical trap arrays on a single device, allowing the imaging of up to 392 animals in quick succession (Fig. 1C). Worms are loaded using a syringe and blunt needle without the need for any additional hardware or extensive training, passively filling the trap channels (∼92% channels filled with a single animal, *n* = 25,000). Filling a trap array in this manner takes approximately 1 min, allowing the loading of an entire device with 392 animals in under 10 min. Once all units have been loaded, the syringe and needle are removed, and the device is mounted on a microscope (movie S1).

Animals can be recovered from the microfluidic devices after image acquisition with nearly 100% efficiency by reversing the flow and collecting the animals off-chip. Moreover, it is possible to selectively label individual animals of interest within the devices using a photoconvertible marker (e.g., *myo-2::dendra*) or any other identifying marker (fig. S1). Therefore, this system can be used for forward genetic screens or other experiments in which rare animals of interest must be retrieved and propagated for further analysis.

Microfluidic devices are fabricated using a custom soft lithography process, enabling the fast, cost-effective production of many nearly identical devices (Fig. 1, C and D, and Supplementary Materials and Methods). This method of fabrication overcomes the inherent device-to-device variation associated with traditional soft lithography techniques (*12*). Using a reproducible casting process, devices are fabricated with a tilt of less than 0.5° and a device-to-device variation of less than 200 μm. The devices are stably mounted in a rigid support frame to ensure reproducible positioning on the microscope stage (Fig. 1C, highlighted in red, and fig. S1D).

### Automated high-throughput image acquisition

**Fig. 2.**
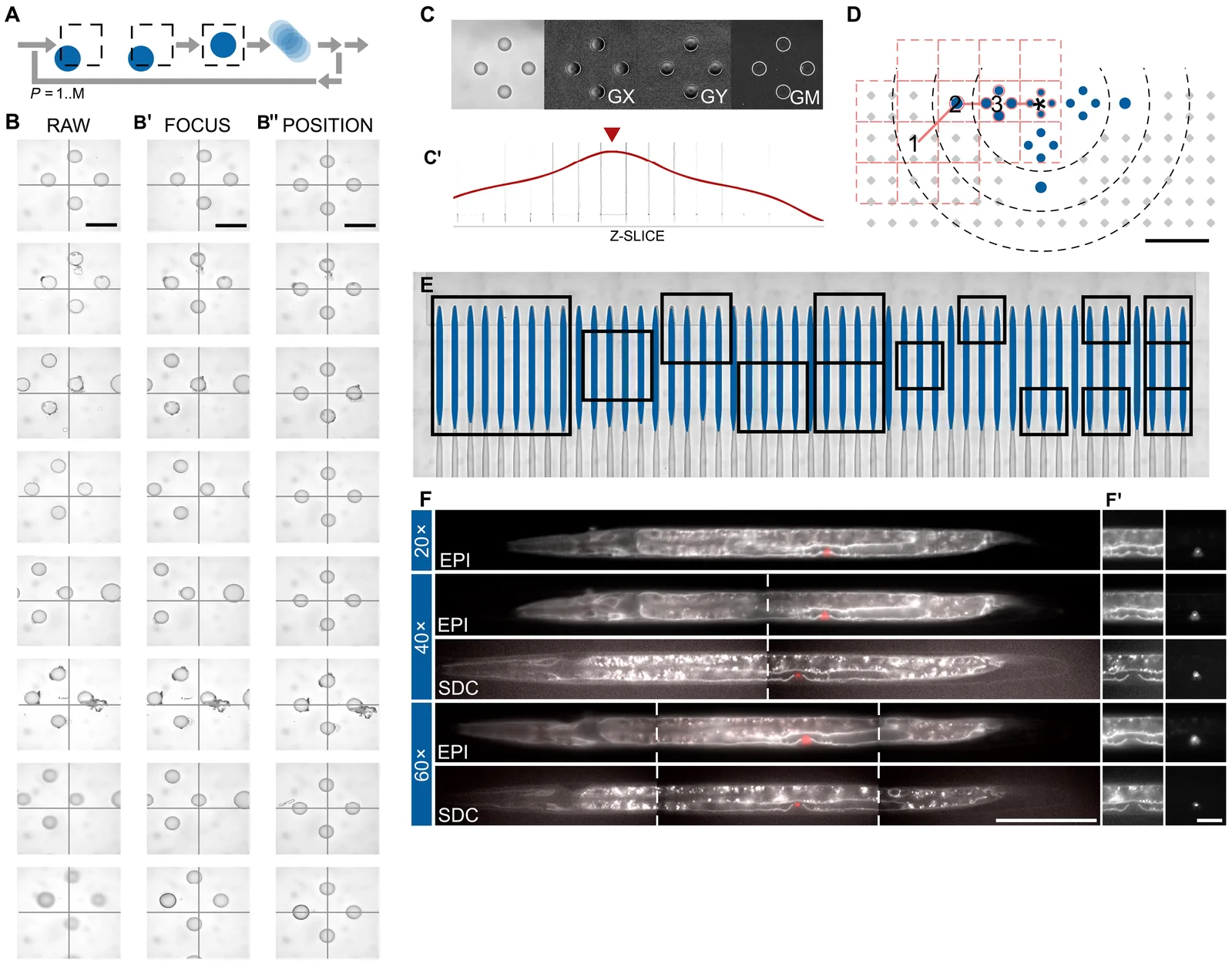
System automation and workflow. (**A**) Workflow overview. The device is placed on the microscope and manually focused on the first unit’s focus and position markers. After manual positioning, the system executes autofocus and position routines, followed by image acquisition on all selected ROIs. If chosen, an additional autofocus is performed. Steps are repeated for all subsequent selected units. (**B**) On-chip markers (circles) without correction. Because of the modified device fabrication protocol used, little change in position or focus is observed from trap array P1 (top) to trap array P8 (bottom). (**B’**) Device markers after focus correction. Identified focus position is propagated along the device, compensating for large potential changes in focus. (**B”**) Position correction applied to each unit. (**C**) In-focus bright-field image, with GX and GY denoting the image gradient in the respective directions, and GM indicating the image gradient magnitude. (**C’**) Overlay of the measured focus curve (red) and the corresponding image. The highest gradient magnitude is observed at the transition from the cover glass to the PDMS channel (arrowhead). (**D**) Automated positioning concept at 40x. Starting from (1), the system identifies circles of different sizes and positions itself at the start point (*). Effectively compensating for position variations up to 1 mm. (**E**) Device- and magnification-dependent ROIs. Example of all selectable ROIs (black squares) in the used L3/4 device at 20× to 60×. (**F** to **F’**) The same animal was imaged automatically at different magnifications (20× to 60×) using an EPI and SDC microscope. If multiple adjacent ROIs are selected (40×/60×), the output images are automatically stitched together (dashed lines). The entire animal is shown (F, overlay AC & VPCs and BM marker), along with separate images for the AC & VPCs and the BM (F’). Scale bars are (A to A”) 100, (D and E) 500, and (F’) 25 μm.

Integration of the microfluidic device into an automated imaging system is achieved with a set of circular positioning markers placed alongside the trap channel array of each unit (Figs. 1A’ and 2 and figs. S1 and S2). These markers are used for autofocusing and automated positioning of the microscope objective relative to the microfluidic trap channel arrays. Autofocusing on these markers and the individual trap channels is done with bright-field image z-stacks (Fig. 2, B and B’, and fig. S1A). Automated positioning is performed once the system has focused, identifying the differently sized circles (Fig. 2C and fig. S2) and iteratively moving from the larger to the smaller circles, which are located at a known distance from the trap channels (Fig. 2C; fig. S2, B to D; and movie S2). The use of these dedicated markers, rather than identifying other device features or the biological sample using computer vision or similar approaches, ensures reliable performance regardless of sample type, magnification, or device structure. Since autofocusing and positioning are performed at the selected imaging magnification, identifying device features at low magnification before image acquisition, as seen in (*3*) is unnecessary (Fig. 2D). This results in a faster and simpler workflow.

High-speed image acquisition takes advantage of the regular trap channel geometry and well-defined worm location on-chip. Animals are imaged according to predefined magnification- and device-specific regions of interest (ROIs), covering specific body regions or the entire animal (Fig. 2E). Images of the separate ROIs are automatically stitched and cropped around the individual animals (Fig. 2F). Using the conditions to record the images shown in Fig. 2 (F and G) with epifluorescence (EPI) illumination (i.e., 35 ms exposure, two colors, and 41 *z*-slices with 0.5 μm spacing) and ROIs covering the entire animal, a throughput of ∼4500 animals per hour at 20×, ∼1475 at 40×, and ∼800 at 60× was achieved. The automated imaging system can also be used with a spinning disk confocal (SDC) microscope or a scanning confocal microscope. Using the conditions shown in Fig. 2 (F and F’) for SDC microscopy [i.e., 500 ms exposure for green fluorescent protein (GFP), 250 ms for mCherry, 41 *z*-slices with 0.5 μm spacing], a throughput of ∼170 animals per hour at 40×, and ∼85 at 60× was achieved. The substantially lower throughput in SDC mode is owed to the longer exposure times required for confocal imaging. Approximately 15% of the time is spent on automatic focus/positioning, 80% on image acquisition, and the remaining time on data formatting and overheads. Data storage is performed in parallel in the background to enhance system performance.

### Targeted RNAi screen for regulators of AC invasion

**Fig. 3.**
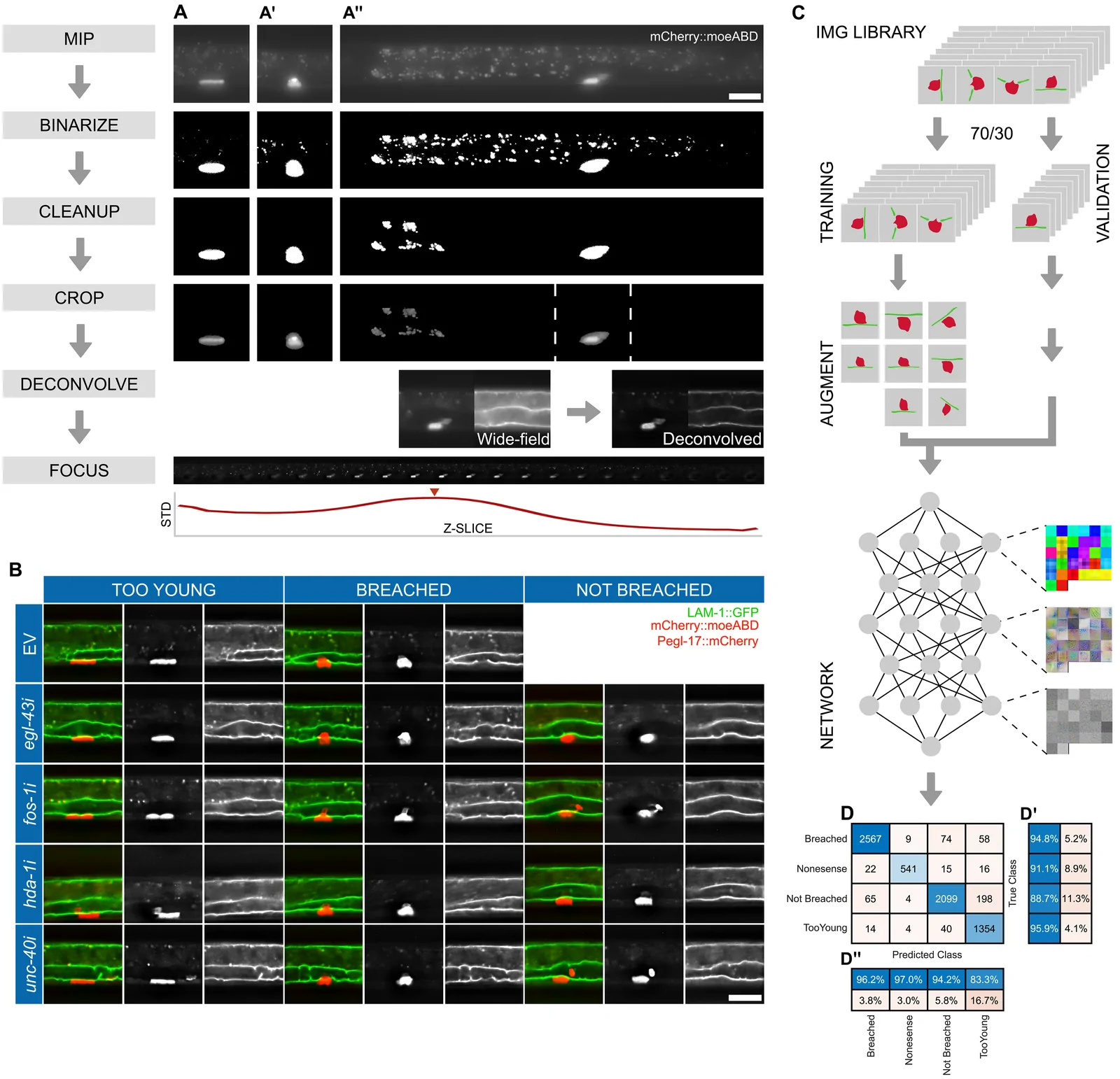
Image analysis workflow. (**A** to **A”**) Preprocessing. The AC & VPCs locations are identified in a maximum intensity projection (MIP) image. The MIP is initially binarized, followed by image cleanup to select the most likely ROI. Last, the stack is cropped, deconvolved, and the in-focus slice is identified based on the image’s standard deviation. The focused slice is then exported as a .png file, with the red and green fluorescent images overlaid after autocontrast. These steps are shown for animals too young to be scored (A), with breached (A’), and nonbreached BMs (A”). (**B**) Images after preprocessing. Training images were acquired from animals grown on EV, *egl-43i*, *fos-1i*, *hda-1i*, and *unc-40i* bacteria. Showing the RGB overlay created by the preprocessing pipeline, along with separate images for the red AC & VPCs markers and green BM marker. (**C**) Concept of transfer learning. Images in the training library are split: 70% network training and 30% performance validation during training. The images in the training library are augmented by rotating, mirroring, resizing, or shearing the original image, with random reapplication of augmentations during each training epoch. These images are then used to retrain an existing convolutional neural network (Inception V3), schematically represented as a series of interconnected layers of nodes. On the right: representative images for three processing layers, recognizing different colors or textures. (**D**) Confusion matrix comparing manually assigned (true class) and network-assigned (predicted class) scores for all images included in the training library. The diagonal indicates the number of images where manual and network scores agree, while the other fields show the number of images where the scores disagree. (**D′**) Shows the true and false-positive rates for each row in (D). (**D”**) shows the positive predictive values and false discovery rates for each column in (D). Scale bars (A and B) are 25 μm.

To identify regulators of AC invasion, we performed RNAi by feeding larvae with dsRNA-producing *Escherichia coli*, which efficiently down-regulates gene expression at the mRNA level (*13*). For example, feeding L1 larvae RNAi bacteria against *egl-43* resulted in a 96% penetrant BM breaching defect at the late L3/early L4 stage (*14*). We thus scored BM breaching in late L3/early L4 larvae fed with RNAi bacteria targeting different candidate genes. We tested genes reported to exhibit a protruding vulva phenotype (Pvl), a defect in dorsal lumen opening, or a depolarization of the actin cytoskeleton in the AC after RNAi knockdown (*9*, *15*) and genes known to be expressed in the AC or VPCs (*16*). These criteria resulted in a list of 193 candidate genes, of which 52 had previously been shown to be necessary for BM breaching, e.g., *egl-43* (*15*, *17*, *18*), *fos-1* (*7*), *hda-1* (*19*), *unc-40* (*20*), and *madd-2* (*15*) (table S1). The 52 genes served as positive controls to assess the method’s efficacy and sensitivity. RNAi experiments were conducted according to the scheme shown in fig. S3A. For each RNAi condition, up to 98 animals were imaged at the late L3/early L4 stage (33 to 36 hours post-L1 starvation), in both the P0 and F1 generations (fig. S3A). BM breaching was scored using three transgenic reporters: *lam-1::gfp (qyIs10)* labeling the BM component Laminin-1, *Pcdh-3>mCherry::moeABD (qyIs50)* highlighting the actin cytoskeleton in the AC (*21*) and *Pegl*-*17>mCherry (zhIs190)* labeling the 1° vulval cells to determine the animals’ stage (Fig. 3, A and C). We used EPI illumination at 40× magnification to image the three markers, recording 41 *z*-slices with 0.5 μm spacing across three channels [differential interference contrast (DIC), GFP, and mCherry] using a centered ROI. Images were acquired across multiple screening rounds, with approximately 40 RNAi conditions imaged per round in the P0 and F1 generations. In addition to the 193 RNAi conditions tested, we included empty RNAi vector-treated (EV) and *egl-43i* animals as negative and positive controls, respectively. In total, we imaged BM breaching in over 40,000 animals at a throughput of ∼1700 animals per hour.

### Neural network–based image classification allows real-time phenotype scoring with high accuracy

Images were preprocessed using a custom-built MATLAB script that automatically detected the ROI containing the AC and VPC markers, identified the in-focus *z*-slice, deconvolved the image stack, and exported an RGB image of the in-focus slice (Fig. 3, A and B; see Supplementary Materials and Methods).

To phenotypically classify the preprocessed images, a neural network was retrained using transfer learning, in which an existing convolutional neural network [here, InceptionV3 (*22*)] was retrained to classify application-specific images. Transfer learning was performed on approximately 7000 manually annotated images across a diverse array of RNAi conditions (EV, *egl-43i*, f*os-1i*, *hda-1i*, and *unc-40i*) (Fig. 3, B and C). These different conditions were selected to capture the potential variability in BM breaching defects. *egl-43i*, *fos-1i*, and *hda-1i* typically exhibited a weak AC signal, with the 1° VPCs migrating toward the vulval midline and forming an invagination under a nonbreached LAM-1::GFP signal. *unc-40i* animals, on the other hand, showed a bright AC signal, but the AC was displaced along the anterior-posterior axis, while the BM remained intact (Fig. 3B and fig. S3B). We classified the observed phenotypes into the following four groups: normal invasion (breached; animal in the late L3/early L4 larval stage showing a breached LAM-1::GFP signal at the site of VPC invagination), defective invasion (not-breached; animal in the late L3/early L4 larval stage showing an continuous, nonbreached LAM-1::GFP signal), animal too young for scoring (too young; animal in the early-mid L3 larval stage showing an nonbreached LAM-1::GFP signal) or nonsense (nonsense; debris, males, etc.) (Fig. 3, A and B). Network training resulted in approximately 94% training and validation accuracy and a validation loss of less than 0.2. Specifically, the trained network identified animals with a breached BM with an accuracy of 94.8%, not-breached BMs with an accuracy of 88.7%, too young animals with an accuracy of 95.9% and images belonging to the nonsense category with an accuracy of 91.1% (Fig. 3D). These results indicate that differentiating between animals too young to score and those with an actual BM breaching defect is most challenging, making it difficult to detect slight delays in BM breaching.

**Fig. 4.**
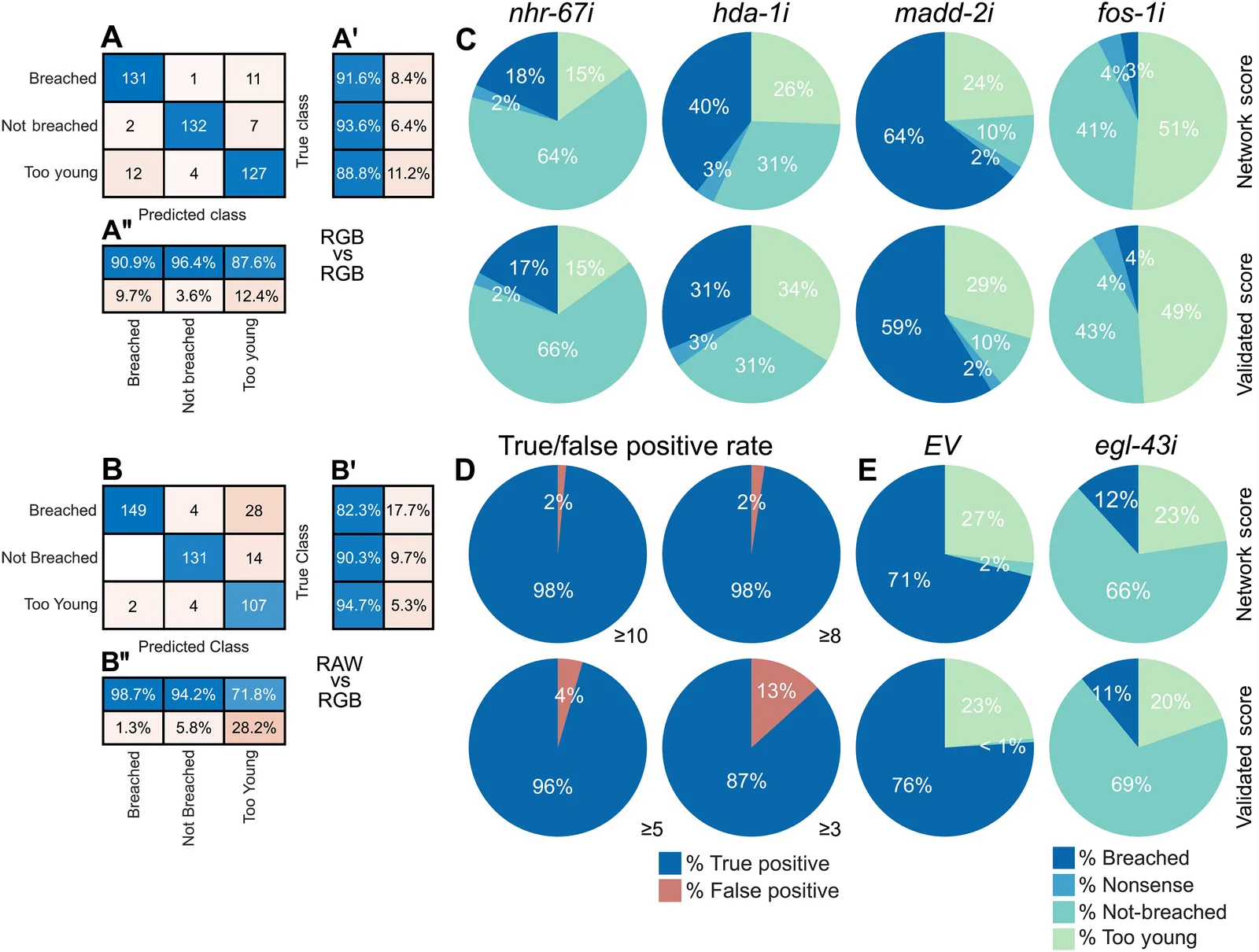
Neural network analysis and validation. (**A**) The confusion matrix compares manually assigned (True Class) and network-assigned (Predicted Class) scores for 500 control images, sampled randomly from EV and *egl-43i*, both using preprocessed RGB images. The diagonal indicates the number of images where manual and network scores match, while the other cells show the number of images with mismatched scores. (**A’**) displays the true-positive and false-positive rates for each row in (A). (**A”**) shows the positive predictive values and false discovery rates for each column in (A). (**B** to **B”**) show the confusion matrix comparing manual scores from the raw 4D dataset with network-assigned scores. (**C**) compares network-assigned scores and manually validated scores for four genes known to control BM breaching (*fos-1i*, *madd-2i*, *hda-1i*, and *nhr-67i*), indicating the percentage of animals in each category. (**D**) shows true/false positive rates for RNAi conditions as a function of the selected cutoff, including all conditions with network scores greater than or equal to the cutoff (≥10, ≥8, ≥5, and ≥3). A lower cutoff results in a higher false-positive rate. (**E**) compares network-assigned scores and manually validated scores for all negative controls (EV, *n* = 1216, across 15 experiment rounds, with 94.33% accuracy) and all positive controls (*egl-43i*, *n* = 570, across eight experiment rounds, with 94.74% accuracy), along with the overall scoring accuracy for each condition.

Network performance was assessed on the control images (i.e., EV and *egl-43i*) acquired during the RNAi screen, effectively scoring performance on known phenotypes in unseen images. Accuracy for these images was 94.0 ± 2.6% for EV animals (15 experiments, *n* = 1268) and 92.5 ± 3.9% for *egl-43i* animals (nine experiments, *n* = 785). Furthermore, we generated a confusion matrix to compare manually assigned scores with network-assigned scores for a subset of 500 EV and EGL-43i images, using either automatically preprocessed images (i.e., cropped and focused RGB images) or the full 4D dataset as input (Fig. 4, A and B). The confusion matrix indicated a good agreement between manual and network scores for the preprocessed RGB images (Fig. 4, A to A”), while manual scores obtained from the raw 4D data compared to the preprocessed RGB images showed a higher discrepancy (Fig. 4, B to B”). However, the differences found are not substantial, indicating that the taken preprocessing steps do not affect obtained scores.

We also assessed network accuracy for other RNAi conditions perturbing BM breaching, two of which had been included in the training library (*fos-1i*: 97.9% accuracy, *n* = 94 and *hda-1i*: 84.9% accuracy, *n* = 93), along with two conditions that had not been used for transfer learning (*madd-2i*: 94.5% accuracy, *n* = 91 and *nhr-67i*: 98.8% accuracy, *n* = 87). Network scores and manually validated scores are shown in Fig. 4C, and representative images in fig. S4A. Moreover, the accuracy for twenty randomly selected RNAi conditions was 92.0 ± 4.6%. Network scores (upper bars) and manually corrected scores (lower bars) are shown in fig. S4B.

To determine the false-positive rate of the screen, we manually validated all network scores to identify conditions that were incorrectly scored as breached or not-breached. In cases of genes with ≥10 not-breached animals recognized by the neural network among up to 98 animals analyzed per condition (minimum 40 scorable animals, average *n* = 67), 1.5% were falsely scored. False-positive rates increased with decreasing numbers of not-breached scores, deviating from the system’s background noise, estimated from the false-positive rate in EV control (∼2.3%). Inversely with higher not-breached score the system’s false-positive rate more closely resembles the background noise (Fig. 4D).

On the other hand, 25 randomly selected conditions (excluding controls) that were predicted not to contain any nonbreached animals were all manually confirmed not to affect BM breaching. On the basis of these data, we estimate a minimum of five not-breached animals among the maximum of 98 animals scored as the threshold for BM breaching defects of interest.

### Automated phenotypic scoring identifies previously unknown regulators of AC invasion

We identified genes of interest from the RNAi screen based on the initial score obtained from the BM breaching classification network described above. Scores derived from network predictions are called network scores. All identified genes of interest were subsequently manually validated, with the resulting scores indicated as validated scores. The penetrance of the BM breaching defects reported in the following was always calculated from the validated scores.

Of the 193 genes tested by RNAi, 52 had previously been reported in the literature to be involved in BM breaching, 76 were reported not to affect BM breaching, nine were ambiguous, and the remaining 57 genes had not been tested (table S1). On the basis of the false-positive rates determined during network validation (Fig. 4), we estimated the image-processing system’s noise to be approximately three false nonbreached counts per 100 animals scored. We thus considered only RNAi conditions with at least five nonbreached animals predicted by the network for further analysis. Of the 193 RNAi conditions tested, 173 could be imaged and scored using our high-throughput imaging and analysis pipeline (tables S1 and S2). The remaining 24 RNAi conditions could not be imaged due to severe developmental defects (e.g., *lit-1i*, *tag-203i*, and *rpn-6.1i*). Eighty of the 173 conditions were imaged and scored in both the P0 and F1 generations. The remaining 93 could only be imaged in the P0 generation due to severe developmental delay or lethality in the F1 generation.

**Fig. 5.**
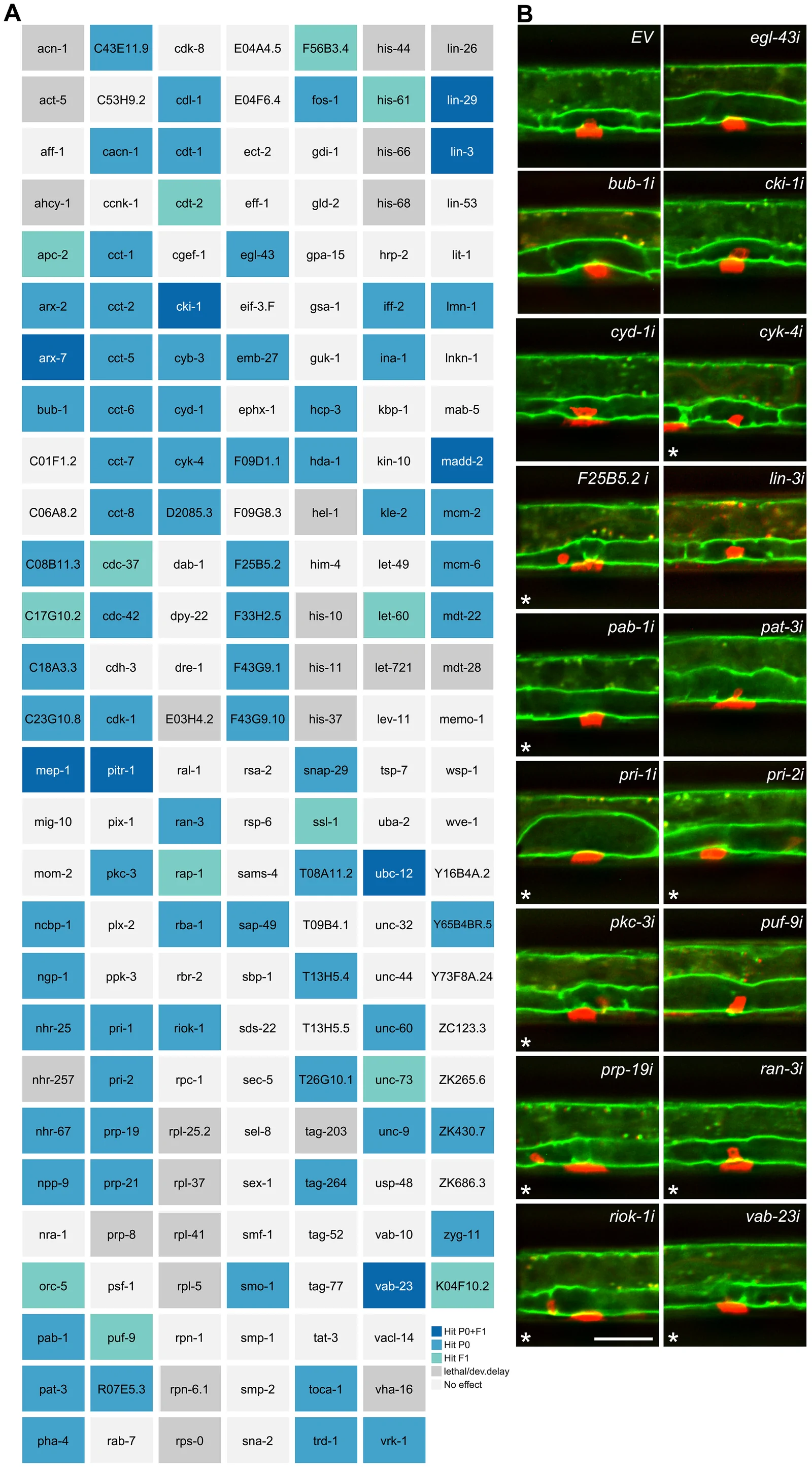
Results of the RNAi screen. (**A**) Hits with an initial network score ≥5 are shown in solid colors. All RNAi conditions that did not affect BM breaching in our experiments are shown in gray. Conditions that were not assessed due to severe developmental delay or lethality are shown in dark gray. (**B**) Sample RGB images for some of the identified hits, along with sample images for all 10 conditions assessed for validation (highlighted with asterisk). Scale bar, (B) 25 μm.

In total, we identified 92 unique hits (Fig. 5 and table S1). Seventy RNAi conditions caused a (i.e., ≥ 5%) BM breaching defect only in the P0 generation, 13 only in the F1 generation and nine had effects in both generations. Overall, the BM breaching defects showed higher penetrance in the F1 than in the P0 generation, indicating that prolonged exposure to the RNAi bacteria increased the penetrance.

Forty-one of the 52 RNAi conditions previously reported to cause BM breaching defects were identified in our screen. Of these, 21 showed a penetrance of ≥10% and thirteen (e.g., *fos-1*, *egl-43*, *lin-3*, *unc-60*, *cct-1*, *cct-2*, *cct-6*, and *cct-*7) exhibited a penetrance of ≥50% not-breached animals (Fig. 5 and table S1). The remaining eleven RNAi conditions predicted to perturb BM breaching were either classified with a low network score (*cdh-3i* 1.3% nonbreached, *gdi-1* 2.7% nonbreached, *let-49i* 1.8% nonbreached, *rsa-2i* 2.7% nonbreached, and *wsp-1i* 1.2% nonbreached), had a validated score of 0% (*ppk-3i*, *sams-4i*, and *usp-48i*) or resulted in severe developmental delay, precluding them from scoring (*guk-1i*, *lit-1i*, and *tag-203i*). These lower-scoring and developmentally delayed conditions are shown in fig. S5.

We identified 35 RNAi conditions that perturbed BM breaching among the 76 genes reported in the literature to have no effect. Of these, 21 showed a penetrance of ≥10% (e.g., *pri-2i* and *mcm-6i*), and three had a penetrance of ≥50% (*prp-21i* and *sap-49i* in the P0 and *puf-9i* in the F1).

Moreover, we identified 10 previously unknown AC invasion regulators among the 57 previously untested RNAi conditions, four of which exhibited a penetrance ≥10% (*pri-1i* and *riok-1i* in the P0, and *K04F10.2i* and *let-60i* in the F1).

Last, we found that six of the nine ambiguous conditions affected BM breaching, with *cdl-1i*, *nhr-*67*i*, and *npp-9i* showing ≥50% penetrance, and *R07E5.3i* and *Y65B4BR.5i* achieving ≥10% penetrance.

Even RNAi conditions that lead to phenotypes not represented in the data used for network training, e.g., displaced AC (e.g., *cdl-1i*), missing VPCs due to induction defects (e.g., *let-60i*, *lin-3i*), depolarized AC (e.g., *cyd-1i*), or malformed gonads (e.g., *cyb-3i*), were identified with high accuracy. Furthermore, animals with missing or very weak AC and VPC signals (e.g., males, *cki-1i*, *T09A5.9i*) were assigned to the nonsense category. Hence, RNAi conditions perturbing both tissues could be identified by an unusually high number of animals assigned to the nonsense category.

We rescreened 10 of the previously unknown invasion inhibitors identified among the genes reported not to affect BM breaching and the previously untested genes (*cyk-4i*, *F25B5.2i*, *pab-1i*, *pkc-3i*, *pri-1i*, *pri-2i*, *prp-19i*, *ran-3i*, *riok-1i*, and *vab-23i*). RNAi against these 10 genes was manually confirmed to cause a BM breaching defect in the P0 generation. We then used strains carrying different markers for genes involved in BM breaching. We qualitatively scored the expression of *fos-1*, *unc-40*, *cdh-3*, and *hlh-2* in the AC, along with the occurrence of multiple ACs, displacement of the AC from the vulval midline, small AC size, and loss of AC polarity. Figure S6 summarizes the observed phenotypes. However, it should be noted that some of the changes occurred with low penetrance and can therefore serve only as an indicator of possible gene function.

In summary, the automated imaging and classification platform identified most genes known to affect BM breaching, as well as several genes not previously known to be involved in the process. For example, RNAi of *pri-1*, which encodes a subunit of the DNA primase complex, resulted in depolarization of the AC and seemingly reduced expression of *fos-1*, *cdh-3*, *unc-40*, and *hlh-2*. Occasionally, *pri-1i* animals contained multiple ACs. RNAi of *pri-2*, which encodes the other DNA primase subunit, caused similar defects (Fig. 5B and fig. S6).

## DISCUSSION

To date, only two high-resolution, high-throughput *C. elegans* microfluidic imaging systems have been available (*1*–*3*). However, the widespread adoption of either system by the research community has been limited, likely due to the complexity of device operation and the need for expensive, specialized equipment. The system presented in this study overcomes these limitations by enabling rapid imaging of thousands of animals per hour using an easy-to-use, inexpensive microfluidic device combined with a fully automated microscope. The method can be used to assess the effect of gene or small molecules on any biological process of interest, using forward or reverse genetic screening approaches, such as RNAi, CRISPRa, or CRISPRi, or drug screens. Moreover, the possibility to label and reliably recover individual animals from the devices enables the conduct of forward mutagenesis screens using high-resolution imaging to isolate and propagate rare mutants of interest.

Thanks to its simple operation, the system can also be used for routine image acquisition, increasing imaging throughput compared to the standard agar pad mounting method.

### Imaging system performance

Our devices, based on the design parameters outlined in Spiri *et al.* (*4*), enable high-resolution imaging using all common imaging modalities (DIC, EPI, and confocal microscopy) without any noticeable drop in image quality compared to conventional agar pad immobilization methods. Furthermore, a custom fabrication protocol enables device fabrication with exceptional precision and reproducibility, while also allowing the easy fabrication of multiple devices at low cost.

Imaging of several thousand animals per hour is achieved through a densely packed trap channel array design, along with a microscope setup capable of high-speed image acquisition. Animals are loaded into the device using a syringe and needle, and the loaded device is then transferred to the imaging microscope. Decoupling worm loading and image acquisition increases throughput, since a new device can be loaded while the previous device is being imaged. Worm preparation and loading will only affect system throughput if images are acquired at a rate greater than approximately 2500 animals/hour, i.e., the maximum number of animals a single person can load in 1 hour. Image acquisition speed is determined by two factors: the time required to automatically position and focus the system on the desired ROI, and the selected imaging parameters (Fig. 1E).

The method’s utility is not limited to a specific developmental stage or biological application. Following the design parameters outlined in Spiri *et al.* (*4*) and the fabrication method outlined above, devices for processes in all larval stages and adult animals can be created and easily integrated into the automated imaging workflow by adapting a few simple geometrical parameters.

### Automated phenotypic scoring

As a proof of principle, we applied the microfluidic screening platform to identify regulators of BM breaching during AC invasion. Given the large amount of data generated (over 40,000 animals were imaged for the primary screen), an automated image analysis pipeline was necessary to classify the phenotypes. The image processing pipeline developed for this study allows fully automated processing and scoring of BM breaching at high throughput (<4 s per animal) and with more than 90% accuracy. High accuracy was achieved not only for RNAi conditions in the network training library, but also for a diverse range of other BM-breaching defects not represented in the training data. Combining this fast and accurate image processing pipeline with the automated microfluidic high-throughput imaging system enables substantially higher imaging throughput than traditional agar pad–based screening and manual scoring. It also delivers substantially more information than plate-based screening approaches (*23*). Last, the presented image processing method, like the image acquisition system, can be applied to score a diverse range of processes by adapting the preprocessing system to detect the desired ROI and retraining the neural network to recognize the phenotypes of interest.

Despite the highly accurate image classification achieved with a retrained neural network, scoring was not perfect. False scores may originate from several sources, such as image preprocessing errors (especially when the in-focus slice was not correctly identified), insufficient network accuracy, or limited phenotypic variation in the training library when scoring phenotypes. Network retraining was performed on images acquired under four RNAi conditions known to affect BM breaching, as well as an EV control. These conditions do not represent all the possible sub-phenotypes. Hence, scoring accuracy inevitably varies for different RNAi conditions. Differentiating animals with an AC invasion delay from those too young to be scored was the most challenging aspect. Animal age is primarily scored on the basis of the appearance of AC and the number of 1° VPC descendants (four 1° cells at the onset of BM breaching). The cytoplasmic localization of the 1° VPC marker used in our study allowed only relatively coarse age scoring, so slight delays in BM breaching could not always be accurately detected. It should be noted, however, that the manual differentiation of slight delays in BM breaching can be difficult even for experienced researchers.

Scoring performance at this early stage of AC invasion could likely be improved by replacing the cytoplasmic marker with a nuclear localized one, such that the number of cells present can be accurately scored.

### RNAi screening identifies previously unknown regulators of BM breaching

With our approach we identified 41 of the 52 RNAi conditions previously reported to affect BM breaching (Figs. 4G and 5 and table S1). The remaining 11 conditions either exhibited a very low BM breaching defect penetrance (approximately 0 to 3%) or failed to develop to the L3 larval stage, such that they could not be imaged and scored. The discrepancy between reported and observed penetrance for some of these RNAi conditions may result from the specific experimental conditions used [e.g., isopropyl-β-d-thiogalactopyranoside (IPTG) concentration and duration of exposure resulting in lethality], the particular RNAi clones used, which can result in less efficient knockdown, and the variable nature of RNAi. Furthermore, RNAi conditions that only caused a slight delay in BM breaching may have been missed when scoring with our trained network.

Twenty-one of the 76 RNAi conditions previously reported not to affect BM breaching reached a penetrance of ≥10%. Also, among the 57 previously untested RNAi conditions, we identified 10 that affect BM breaching, which is an unexpectedly large number. The relatively high frequency of hits could be due to several factors: (i) We imaged up to 96 animals per RNAi clone, scoring substantially more animals than typically achieved using agar pad–based immobilization. This could lead to a more sensitive detection of low-penetrance phenotypes. (ii) We performed our screen in an *rrf-3* RNAi-hypersensitive background to increase the penetrance of defects (*24*). (iii) dsRNA expression in the RNAi bacteria was first induced in the liquid cultures (1 mM IPTG for 4 hours at 37°C), and then again on the nematode growth medium (NGM) plates, potentially resulting in a more penetrant RNAi effect compared to single induction on plates (*25*). (iv) Last, we considered all conditions resulting in an uninterrupted LAM-1::GFP signal as a BM breaching defect. Certain conditions resulting in nonbreached BMs have indirect effects and may not have been considered as an actual BM breaching defect by others. For example, *lin-3i* results in a BM breaching defect due to the absence of 1° VPC induction and thus a lack of the guidance cue secreted by the 1° VPCs (*5*).

Rescreening of 10 newly identified regulators of BM breaching with different AC reporters revealed a variety of underlying defects, including depolarization of the actomyosin cytoskeleton, displacement of the AC from the vulval midline, and reduced expression of transcriptional regulators of AC invasion. The identification of *pri-1* and *pri-2* as regulators of BM breaching is of particular interest. The *C. elegans pri* genes encode the two subunits of the DNA primase complex, which synthesizes short RNA primers during DNA replication. Although the AC is permanently arrested in the G_1_ phase and never replicates its genome, we have previously reported that components of the DNA prereplication complex (pre-RC) are necessary for AC invasion (*26*). Considering these findings, the primase complex may act as a downstream target of the pre-RC in the AC, inducing or maintaining the invasive cell fate. The recruitment of primase complex by the pre-RC may affect chromatin assembly and thereby regulate gene expression near DNA replication origins (*27*).

### Limitations of the method

Despite the numerous advantages, several limitations should be considered when using our microfluidic screening platform. Manual worm preparation and loading limit throughput to approximately 2500 animals per hour. This can be mitigated by loading multiple devices in parallel, but developing an automated loading scheme will be essential to achieve higher throughput in future experiments. Since head/tail orientation cannot be controlled during worm loading, imaging specific ROIs at either extremity (i.e., the pharynx or tail) will require capturing images on both sides of the trap channel, resulting in extraneous data. While our automated imaging system can acquire these large ROIs much faster than using agar pads, storing and analyzing these large datasets may be challenging.

Furthermore, all animals imaged were grown in synchronized cultures for optimal loading efficiency. Working with nonsynchronized populations is possible, but this may result in acquiring many empty channels or channels filled with animals at undesired developmental stages, thereby increasing the collection of “empty” data. For example, AC invasion occurs at the transition from the L3 to the L4 stage and therefore cannot be scored in younger animals. Likewise, scoring older animals should be avoided because invasion delays may be missed, and other factors, such as mechanical expansion of the developing vulva, could mask subtle invasion defects. Hence, RNAi conditions that affect developmental timing, resulting in many younger or older animals, need to be rescreened for reliable scoring. This issue is not unique to AC invasion and may occur with other processes that require scoring at a specific stage.

Currently, the imaging platform is optimized for *C. elegans* larvae and adults. However, it should be possible to adapt it to *C. elegans* embryos, or other small animal models, such as Zebrafish and *Drosophila* larvae, for similar applications.

## MATERIALS AND METHODS

### *C. elegans* alleles and transgenes

For standard worm maintenance, animals were grown at 20°C on NGM [17 g of agar, 2.5 g of NaCl, 2 g of tryptone, 25 ml of KPO buffer (108.3 g of KH_2_PO_4_ and 35.6 g K_2_HPO_4_ to 1 liter of H_2_O), 1 ml of 1 M MgSO_4_, 1 ml of 1 M CaCl_2_, and 1 ml of cholesterol (5 mg/ml) in ethanol (add after autoclavation), add H_2_O to 1 liter] plates seeded with *E. coli* OP50 as described (*28*). The *zhIs190*, outlining all 1° VPCs, was generated using MosSci protocol (*29*). For more details, see the “Plasmid construction and single-copy genome insertion” section below. Table S2 contains a list of strains, along with their corresponding genotypes. The strain AH6296 was generated directly through injection and transgenesis, as described in “Plasmid construction and single-copy genome insertion” section. The strain AH6296 was generated through crosses and subsequently checked for homozygosity by polymerase chain reaction genotyping.

### RNAi plate preparation

For all RNAi conditions used in this study, bacterial clones were picked from the Ahringer RNAi feeding library and verified by sequencing. Before each experiment, up to 40 RNAi bacterial clones were streaked on LB agar plates (5 g of yeast extract, 10 g of peptone, 10 g of sodium chloride, and 12 g of agar to 1 liter of H_2_0) supplemented with ampicillin (0.1 mg/ml final concentration), from frozen stock and grown overnight at 37°C. Three milliliters of LB medium (5 g of yeast extract, 10 g of peptone, and 10 g of sodium chloride in 1 liter of H_2_0) supplemented with ampicillin (0.1 mg/ml final concentration) and tetracycline (0.01 mg/ml final concentration) was inoculated with bacteria from the stock LB plates and grown at 37°C overnight. Following this initial growth period, an additional 3 ml of LB medium supplemented with IPTG (1 mM final concentration), ampicillin (0.1 mg/ml final concentration), and tetracycline (0.01 mg/ml final concentration) was added to the overnight culture and bacteria were grown for another 4 hours to induce dsRNA synthesis (*30*). After this second growth phase, bacteria were seeded onto RNAi NGM plates [17 g of agar, 2.5 g of NaCl, 2 g of tryptone, 25 ml of KPO buffer (108.3 g of KH_2_PO_4_ and 35.6 g K_2_HPO_4_ to 1 liter of H_2_O), 1 ml of 1 M MgSO_4_, 1 ml of 1 M CaCl_2_, and 1 ml of cholesterol (5 mg/ml) in ethanol (add after autoclavation), add H_2_0 to 1 liter], supplemented with ampicillin (0.1 mg/ml final concentration) and IPTG (1 mM final concentration), and allowed to grow on plate, at room temperature (RT) for at least 24 hours. For each RNAi condition, six plates were prepared containing the respective bacteria and stored at 4°C (for up to 5 days) before worm seeding.

### Worm preparation

For RNAi experiments, animals were seeded on RNAi plates prepared as described in the RNAi plate preparation section. Worms were synchronized by bleaching gravid adult hermaphrodites (*31*). The collected embryos were pelleted by centrifugation (1300*g* for 1 min) and washed with fresh M9 buffer (3 g of KH_2_PO_4_, 6 g of Na_2_HPO_4_, and 5 g of NaCl in 1 liter of H_2_O, with 1 ml of 1 M MgSO_4_ added after autoclaving) four times. Embryos were left to hatch overnight in M9 buffer. After overnight incubation, arrested L1 larvae were passed through a 10-μm filter (PluriStrainer Mini 10 μm, PluriSelect, USA) to remove debris and unhatched embryos. They were then washed twice with fresh buffer and seeded onto plates previously coated with different RNAi bacteria (four plates per condition). Animals were seeded at a density of 350 to 400 animals per plate. After approximately 33 to 36 hours at 20°C, P0 animals were collected from two plates and loaded into the device, imaging the late L3/early L4 larval stage. Animals on the remaining two plates were left to grow for an additional 24 to 36 hours, allowing them to reach adulthood, after which they were individually bleached. The overnight-arrested F1 L1 larvae were again passed through a 10 μm filter (PluriStrainer Mini, 10 μm, PluriSelect, USA) and seeded onto plates containing the same RNAi bacterial clone (two plates per condition). F1 animals were again grown for approximately 33 to 36 hours before imaging. For both P0 and F1, up to 98 animals were imaged and scored. Conditions resulting in an excessive number of young animals were rescreened separately (less than 40 scorable animals).

### Plasmid construction and single-copy genome insertion

For plasmid pSS44 (*Pegl-17::mcherry::unc-54 3′UTR* in pCFJ151): The *egl-17* promoter mk84-148 (*32*), along with the pCFj151 backbone, was amplified using the primer pair OAF309/OAF172. The *mcherry::unc-54 3′UTR* containing the complementary part of the pCFJ151 backbone was amplified with the primer pair OSS281/OAF169. The plasmid pAF50 (*33*) was used as a template for both fragments. The two fragments were subsequently fused by Gibson assembly cloning (*34*). pSS44 was used to generate a single-copy insertion in *oxTi444[ttTi5605+NeoR(+)+unc18(+)]; unc-119(ed3)* on LGIII using the MosSci protocol (*29*). Primers used in this study: OAF169: GAGTCCAACCCGGTAAGACACGAC; OAF172: GTCGTGTCTTACCGGGTTGGACTCAAGACGATAGTTACCGGATAAG; OAF309: GAGCTCGGTACCCTCCAAGCAAG; and OSS281: CTTGGAGGGTACCGAGCTCATGGTCTCAAAGGGTGAAGAAGATAAC.

### Microfluidic device fabrication

Microfluidic devices were fabricated using standard photolithography (*12*) and a modified soft lithographic protocol. In brief, master molds were fabricated on silicon wafers (Si-Wafer 4P0/>1/525 ± 25/SSP/TTV < 10, Siegert Wafer, Germany) using SU8 photoresist (GM1050 and GM1060, Gersteltec, Switzerland). Layers of different heights were fabricated consecutively on the same wafer and aligned to each other using a mask aligner (UV-KUB3, Kloe, France). Layers were fabricated with heights of 5 and 20 μm (for low and worm sections, respectively). The wafer was treated with chlorotrimethyl silane (Sigma Aldrich, Switzerland) vapor before polydimethylsiloxane (PDMS) elastomer casting, to prevent adhesion of the PDMS to the wafer and SU8 structures.

A first PDMS mold (Elastosil RT601; ratio 10:1, Wacker, Germany) was fabricated directly from the SU8 master mold and cured at RT. The PDMS was then removed from the wafer, roughly cut to size and placed aligned to the markers on the milled surface of a casting dish. Once aligned, the dish was filled with PDMS and left to cure at RT. After ∼24 hours, the silicone mold was carefully removed from the dish and placed in a vacuum desiccator for 1 to 2 hours. Once degassed, the mold was removed and filled with ∼15 g of polyurethane (PU) mix (Smooth-Cast ONYX/1 Slow; ratio 1:1 by volume, Kaupo AG, Germany) and left to harden. Finally, PDMS devices were cast by filling an assembly consisting of a PU master mold, a support frame and a lid with PDMS (∼10 g per assembly), and cured at RT. Once cured, the frame with the attached PDMS structure was removed from the assembly, access holes punched (16G Catheter Punch, Syneo, USA), and the device bonded to cover glass (75 × 25 mm cover glass with selected thickness 0.17 ± 0.01 mm, Hecht Assistant, Germany) using an air plasma (Zepto Plasma cleaner, Diener, Germany).

### Device operation and worm preparation

For each RNAi condition, animals were washed off two RNAi plates using S-Basal buffer [5.85 g of NaCl, 1 g of K_2_HPO_4_, 6 of gKH_2_PO_4_, 1 ml of cholesterol (5 mg/ml in ethanol), H_2_O to 1 liter; sterilized by autoclaving; Sigma-Aldrich] supplemented with 1 weight % Pluronic F127 (Sigma-Aldrich) and washed trice with fresh S-Basal + F127 to remove any debris. Worms were loaded into the device using a 1-ml syringe equipped with a blunt needle (IP718150-45, 18G, 1.5″ blunt needle, Gonano Dosiertechnik GmbH, Austria). Washed worms were gently sucked into a blunt needle, and the needle was inserted into the device inlet. Animals were loaded by applying pressure to the syringe plunger while observing them through a dissection microscope. Once all channels were filled, the needle was removed, and the device was flushed with anesthetic solution (100 mM tetramisol hydrochloride, Sigma-Aldrich, filled into a second syringe with a needle). Alternatively, the tetramisole can be added to the worm suspension before loading. Last, the device was mounted on the microscope, and images were acquired.

### Image acquisition

Images were acquired on an EPI microscope (DMRA, Leica, Switzerland, equipped with a dual-band beam splitter F58-019, AHF Analysentechnik, Germany), equipped with two sCMOS camera (ORCA Flash4.0-LT+, C11440-42U30, Hamamatsu, Japan) connected to an image splitter (TwinCam, Cairn Research, UK, with dual-band beamsplitter F38-596, and emission filters F47-525, and F47-632 AHF Analysentechnik, Germany), a fluorescence light source (Spectra, Lumencor, USA), and bright-field LED (B200-RGB, ScopeLED, USA), and z-stacks acquired using a piezo objective drive (MIPOS 100 PL SG, Piezosystems Jena, Germany).

Images were acquired using a 20× air immersion lens (HC PL APO 20×/0.80, Leica, Switzerland; sample images Fig. 2, F and F’), a 40× water immersion lens (HC PL APO 40×/1.10 W CORR CS, Leica, Switzerland; RNAi screen), a 40× (HCX PL APO 40×/1.30 Oil, Leica, Switzerland; or UPlanFL 40×/1.30 Oil, Olympus, Switzerland; sample images Fig. 2, F and F’), a 60× (UPlanAPO 60×/1.40 Oil, Olympus, Switzerland; sample images Fig. 2, F and F’), or a 63× oil immersion lens (HCX PL APO 63×/1.32 to 0.60 Oil, Leica, Switzerland; sample images Fig. 2, F and F’). Other lenses may be used, depending on the application, with air, water, or silicon oil immersion preferred due to the low viscosity of the immersion medium. Image acquisition was controlled through a custom-written MATLAB script (MATLAB 2023b, MathWorks, USA) and a custom-built microcontroller (Arduino Mega 2560, Arduino, Italy), which coordinates the fluorescence and bright-field LEDs, piezo, and cameras.

### Automated image acquisition workflow

During operation, a device is placed onto the microscope stage, and markers adjacent to the first selected trap array are roughly identified manually. Once coarsely positioned, the system automatically focuses on the markers and positions itself at the start point. This starting point is located at a known distance from the first imaging ROI, with all subsequent ROIs following a geometric pattern using a simple set of calibration parameters. If selected, an additional autofocus step is executed, compensating for all residual changes in focus, followed by acquisition of the desired multicolor image stack (Fig. 2D). Positioning on all selected, subsequent trap channel arrays (user-selectable) is achieved fully automatically with no need for manual intervention.

Autofocusing is achieved on bright-field image *z*-stacks. In focus is defined as the transition from the cover glass to the PDMS channel. To achieve this, the Sobel gradient magnitude is calculated, with the slice showing the highest standard deviation selected as in-focus (Fig. 2, B and B’). Autofocus at each imaging ROI is achieved solely using the device channels and independent of the animals loaded into the device, resulting in a reliable start point for image acquisition. Automated positioning is performed once the system is focused, identifying the differently sized circles in bright-field images (Fig. 2C and fig. S2). The system preferentially identifies the smallest four circles, with the imaging start point defined as the midpoint of all four circles (fig. S2A). However, if the smallest circles are not visible in a single FOV, the system will iteratively identify the different sets of larger circles in tile images, moving from the larger to smaller circles (Fig. 2C and fig. S2, B to D). All autofocus and positioning steps are carried out at the selected imaging magnification. The identification of device features at low magnification, separate from image acquisition, as seen in other systems (*3*), is therefore not necessary, resulting in a quicker and simpler workflow.

### Image analysis

All image analysis was carried out in MATLAB (MATLAB 2023b, MathWorks, USA) using a custom-built processing pipeline (Fig. 3). Acquired image *z*-stacks were loaded, and the ROI was identified using the AC and VPC markers, identifying the brightest suitable spot in a maximum intensity projection of the mCherry signal after binarization and image cleanup. Threshold, minimum/maximum object size, and minimum average intensity, used during binarization and cleanup, were initially determined empirically to minimize the number of animals lost during preprocessing, while excluding all channels not containing an animal. Images were then cropped to a 376 × 376 pixel square around the most likely AC/VPC location, followed by deconvolution using the YacuDecu (*35*) implementation of CUDA-based Richardson Lucy deconvolution. The in-focus slice was identified using the same red fluorescent markers, with in-focus defined as the slice with the highest intensity SD, assuming that the green fluorescent marker will be in focus at the same *z*-plane as the red fluorescent marker. Once cropped, focused, and deconvolved, an RGB image combining the two markers was saved (Fig. 3, B and C). RGB images were exported and stored as .png images, with an autocontrast applied to all images by saturating the bottom and top 1% of all pixel values before saving. Autocontrast was used to ensure uniform image intensity for all 40,000 animals imaged, compensating for changes in fluorescence intensity associated with animal age, stochastic variation in fluorescence reporter expression, or unevenness in sample illumination. Preprocessed images were then either stored for use in neural network training (training set) or directly fed into the trained network for image classification (RNAi screen).

Image classification was performed using a convolutional neural network [InceptionV3 (*22*)]. Network training was likewise performed in MATLAB (Fig. 3D), supplying between 1000 and 2500 manually scored images of successful AC invasion (breached), unsuccessful AC invasion in animals of suitable age (late L3/early L4 larval stage) (not-breached), animals too young for AC invasion to occur (mid L3 stage or younger) (too young), and images showing gut granules or contaminants not easily removed by the preprocessing pipeline (nonsense) (Fig. 3C). Training images were acquired in animals grown on empty vector, *egl-43i*, *fos-1i*, *hda-1i*, and *unc-40i* all of which are known to interfere with BM breaching. Empty vector and *egl-43i* were also imaged as negative and positive controls throughout the RNAi screen to assess false positives and RNAi efficacy, respectively. Classification results obtained during both network training and final scoring were manually validated.

## Supplementary Materials

### The PDF file includes:

Figs. S1 to S6 Legends for movies S1 and S2 Legends for files S1 to S4 Legends for tables S1 and S2 Extended Materials and Methods References
