# Supplementary material for: An automated high-resolution screening platform identifies regulators of anchor cell invasion in C. elegans
Source: Sci Adv. 2026 Sep 18;12(38):eaef6546. doi: 10.1126/sciadv.aef6546 (PMC13588180; doi:10.1126/sciadv.aef6546)
Supplement: Supplementary file 1 [file sciadv.aef6546_sm.pdf]

Science Advances
AAAS

Supplementary Materials for

An automated high-resolution screening platform identifies regulators of anchor cell invasion in *C. elegans*

Simon Berger *et al.*

Corresponding author: Simon Berger, simon.berger@mls.uzh.ch

Sci. Adv. **12**, eaef6546 (2026)
DOI: 10.1126/sciadv.aef6546

The PDF file includes:

Figs. S1 to S6
Legends for movies S1 and S2
Legends for files S1 to S4
Legends for tables S1 and S2
Extended Materials and Methods
References

Other Supplementary Material for this manuscript includes the following:

Movies S1 and S2
Files S1 to S4
Tables S1 and S2

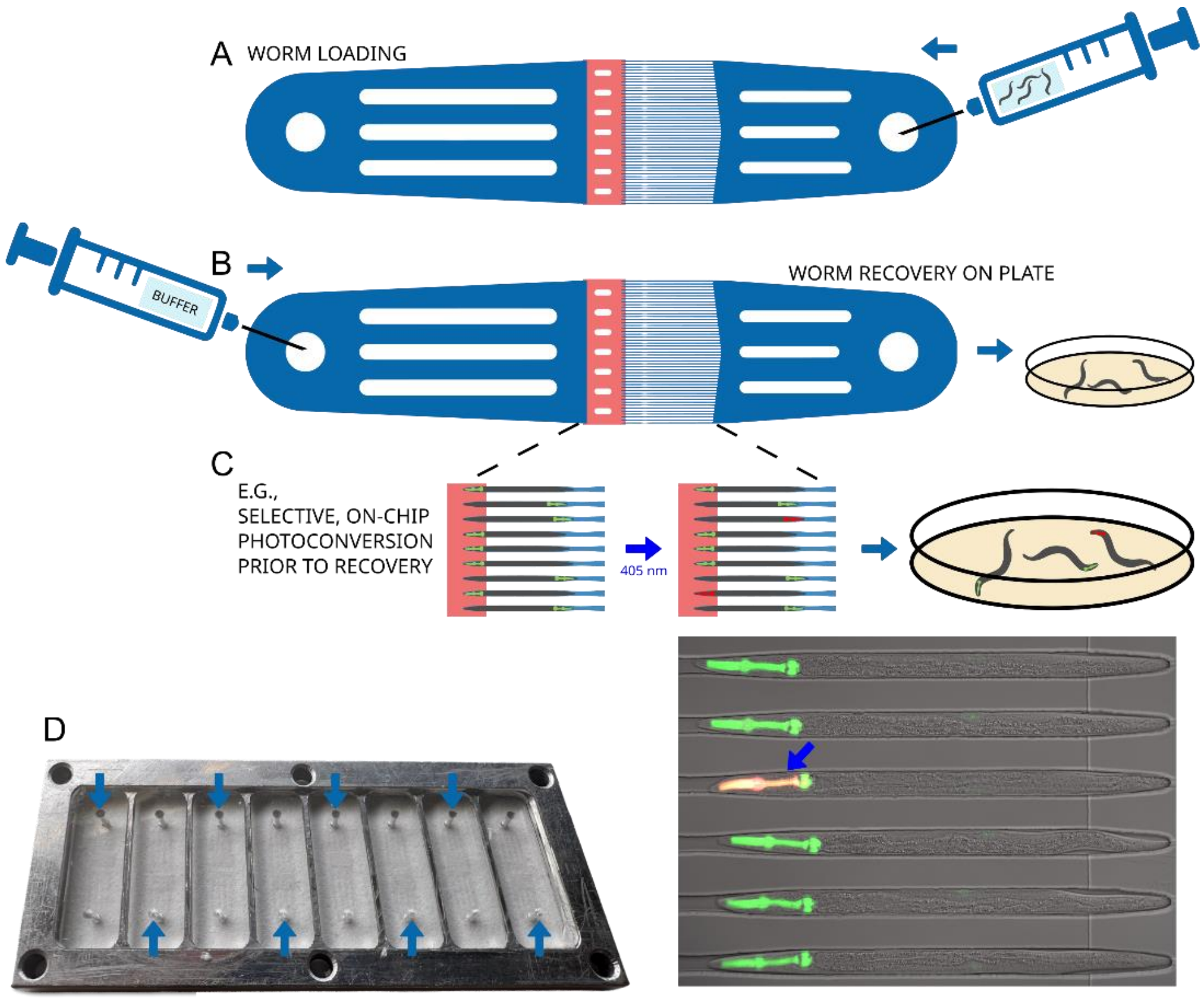

Fig. S1 Device loading and recovery of selected animals. (**A**) Worms are introduced through the worm inlet (right) using a syringe filled with S-Basal buffer and a blunt needle. Animals washed off a plate are carefully drawn into the needle and gently maneuvered toward the trap channel array (arrow pointing left) by pushing and pulling on the syringe plunger. (**B**) Once imaged, all animals in a device unit can be recovered by flushing the unit with clean buffer from the device outlet (left), with a short piece of tubing attached to the outlet side, collecting all animals on an empty NGM plate. (**C, C''**) Specific animals of interest can be labeled inside the device, for example, using a photo-convertible headmarker like *Pmyo-2::Dendra*, selectively switching the marker from green (left) to red (right) for an animal of interest. Magnified overlay of fluorescence images highlighting the shift from green to red for the third worm (arrow). Converted animals can then be identified on the NGM plate and picked for further propagation. (**D**) Image of a device attached to the aluminum support frame. Arrows indicate the inlet through which animals are loaded into the device (**A**) or from which they are recovered (**B**).

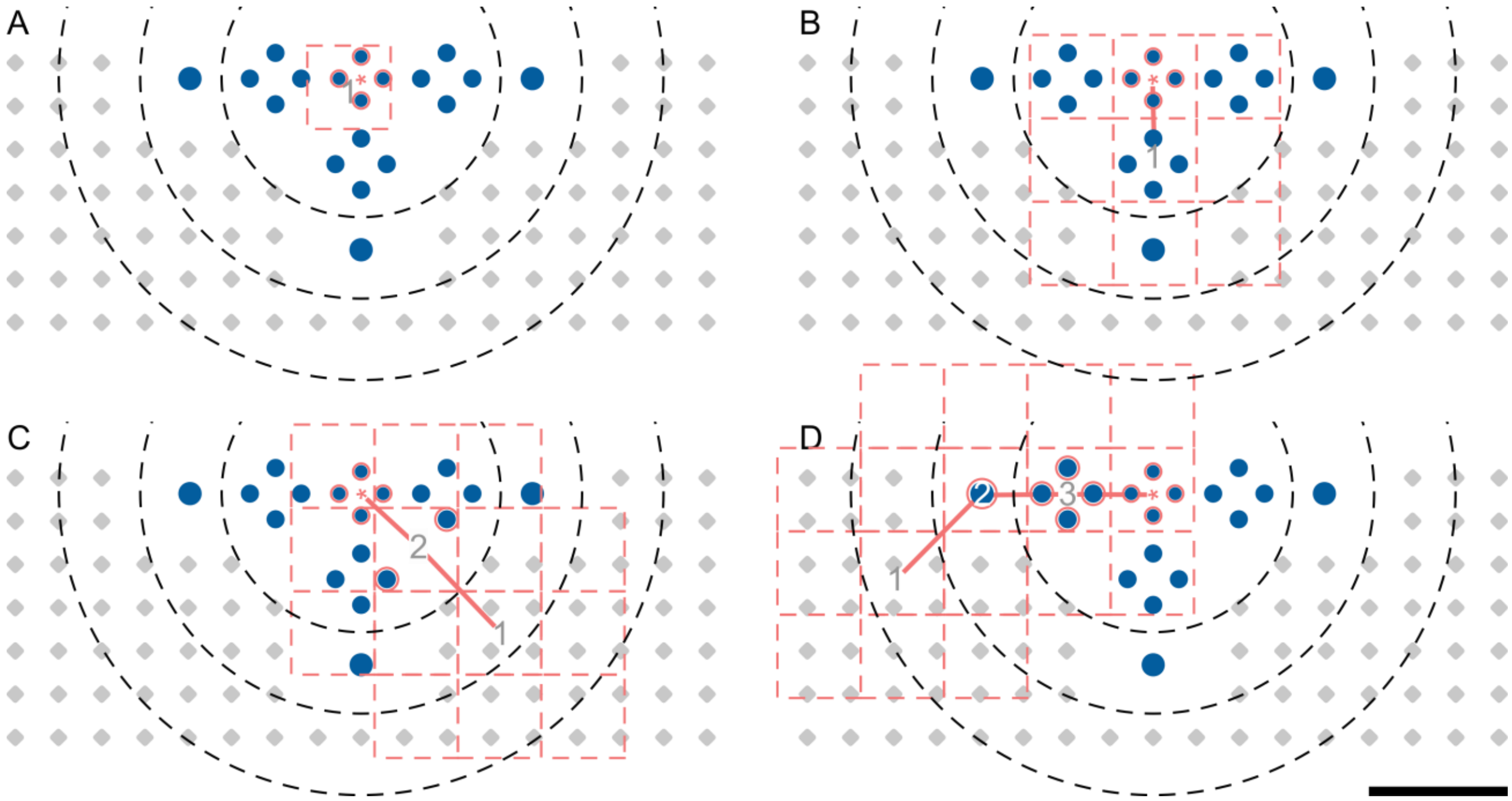

Fig. S2 Auto-positioning scenarios at 40X magnification. (**A**) Microscope initial position (1) close to final position (*) (less than 300 µm). All four smallest circles (highlighted in red) are within the initial field of view (dashed square). (**B**) Microscope initial position (1) one step away from the final position (*) (about 300 µm). None of the smallest circles are visible in the first field of view, resulting in the acquisition of a 3x3-tile image in which the smallest circles can be detected (highlighted in red). (**C**) Microscope initial position (1) two steps away from the final position (*) (approximately 600 µm). None of the small circles are visible in the initial field of view or the first 3x3-tile image. Two medium-sized circles (highlighted in red) are visible in the tile image resulting in acquisition of a second 3x3-tile image at the average position of all detected circles (2), in which the smallest circles are visible. (**D**) Microscope initial position (1) three steps away from the final position (*) (about 1000 µm). Neither the smallest nor medium-sized circles are visible in the initial image or the first 3x3-tile image. The largest circle is therefore detected (2), followed by the acquisition of another 3x3-tile image identifying the medium-sized circles (3), and finally, a last 3x3-tile image in which the smallest circles are visible. Dashed circles indicate distances of 500 µm, 750 µm, and 1000 µm from the center. Scale bar 500 µm.

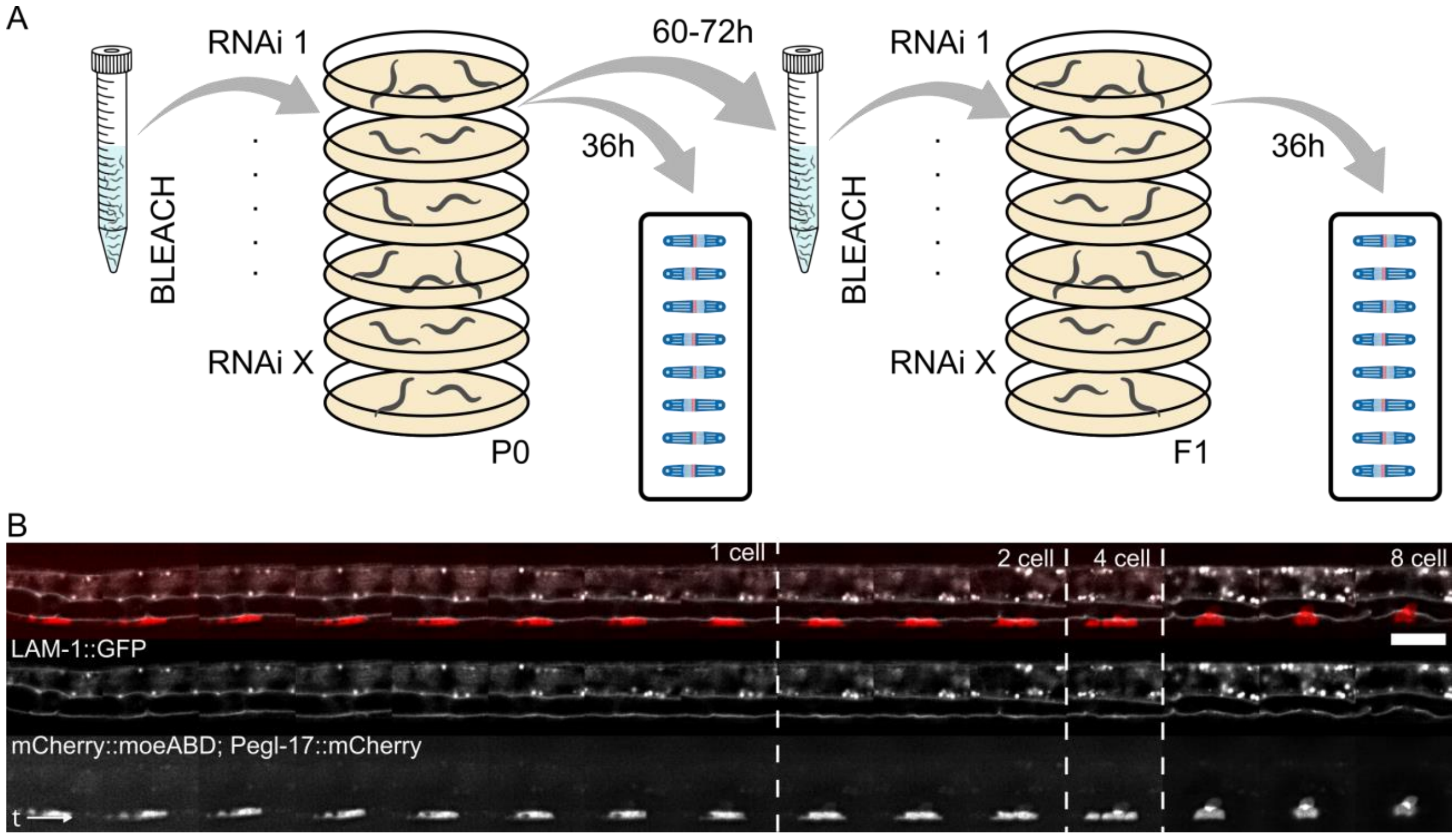

Fig. S3 Experimental Workflow and Marker Progression. (**A**) Synchronized worms are transferred onto RNAi plates seeded with one of the 193 RNAi bacterial clones (six plates per condition, approximately 400 animals per plate). The animals are left to grow for 33-36 hours on the plates, after which worms are washed off four plates and loaded into devices for imaging. The remaining animals continue to grow for an additional 24-36 hours, then adult animals are washed off each plate, and the F1 generation is synchronized by bleaching. These animals are then left to hatch overnight and replated on the same RNAi plates. After another 33-36 hours, F1 animals are washed off the plates and loaded into devices for imaging. (**B**) Marker progression. Shown is a single animal developing from mid L2 stage (single 1° fated VPC visible), into the L3 (2/4 cell) and early L4 stage (8 cell), indicated by the *Pegl-17::mCherry* marker shown in red (overlay top) and bottom panel. At the transition from L3 to L4 stage a hole appears in the BM, yielding a visible interruption of the LAM-1::GFP signal gray (overlay top and middle) and middle panel. Simultaneously the AC indicated by the mCherry::moeABD (red, overlay top and bottom panel) polarizes toward the VPC and breaches the BM. Scale bar 25 μm.

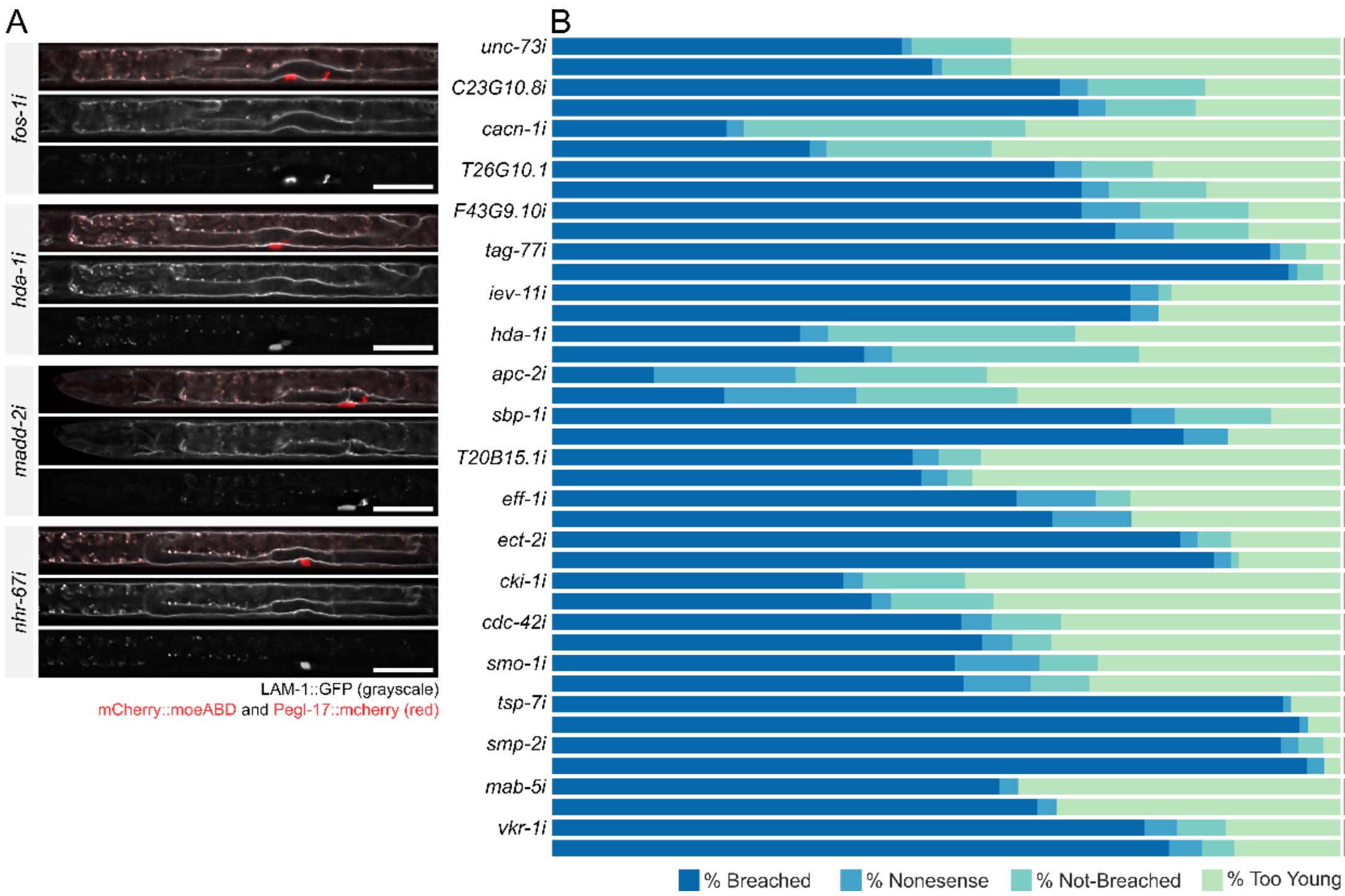

Fig. S4 Network Validation. (**A**) Representative, full images of the four genes known to inhibit BM breaching, assessed in **Fig. 4C**. Each image shows an overlay of the LAM-1::GFP (BM, gray) marker and the *mCherry::moeABD* and *Pegl-17::mCherry* (AC & VPCs, red), displayed at the top, with the individual markers shown below. (**B**) Comparison of network assigned scores (top bar) and manually validated scores (bottom bar) for 20 randomly selected RNAi conditions. Each colored segment indicates the percentage of animals assigned to the four scoring categories. Scale bars (**A**) are 50 µm.

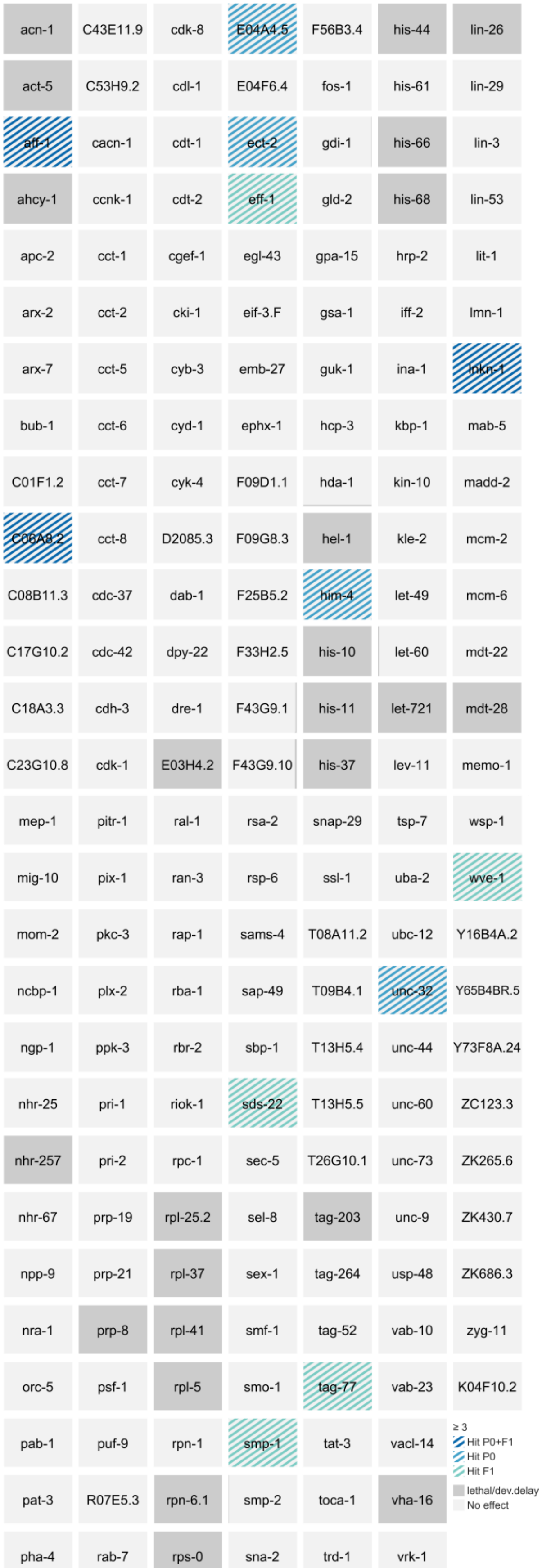

Fig. S5 Additional hits obtained with an initial network score of ≥ 3 shown in hatched colors. All RNAi conditions not assessed due too severe developmental delay or lethality are shown in gray.

Gene	(1) multiple ACs	(2) depolarized AC	(3) displaced AC	(4) small AC	(5) Reduced or absent expression of:			
					(a) fos-1	*(b) cdh-3*	*(c) hlh-2*	*(d) unc-40*
cyk-4i	one strain	several strains	one strain	one strain	one strain	one strain	one strain	one strain
F25B5.2i	not observed	one strain	several strains	not observed	not observed	one strain	one strain	not observed
pab-1i	one strain	not observed	one strain	one strain	not observed	one strain	not observed	one strain
pkc-3i	one strain	one strain	one strain	not observed	one strain	not observed	one strain	not observed
pri-1i	one strain	several strains	not observed	not observed	one strain	one strain	one strain	one strain
pri-2i	one strain	several strains	not observed	not observed	one strain	one strain	one strain	not observed
prp-19i	one strain	several strains	one strain	one strain	not observed	one strain	one strain	not observed
ran-3i	not observed	several strains	not observed	not observed	not observed	one strain	one strain	not observed
riok-1i	one strain	several strains	one strain	not observed	not observed	one strain	one strain	not observed
vab-23	one strain	one strain	several strains	not observed	not observed	one strain	one strain	one strain
empty vector	not observed	not observed	not observed	not observed	not observed	not observed	not observed	not observed

several strains	one strain	not observed

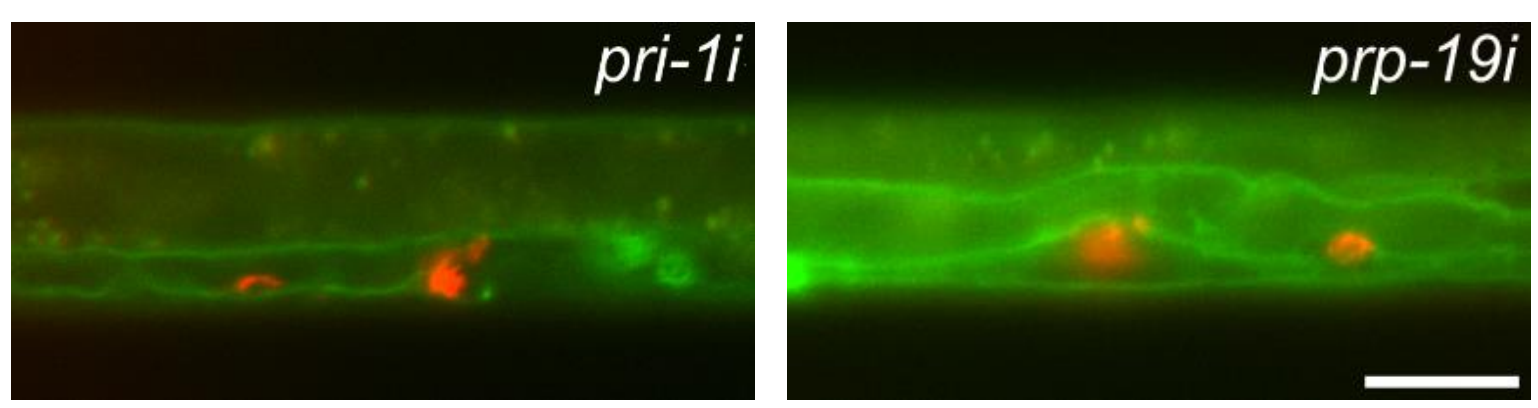

Fig. S6 Re-screening of ten selected candidates. Breaching was scored using *qyIs10[lam-1::gfp; unc-119(+)] IV* or *qyIs127 [lam-1p::lam-1::mCherry + unc-119(+)]* markers, AC location, size, number, and polarity was scored using *qyIs50[cdh-3>mCherry::moeABD]V*. The remaining phenotypes were scored using gene specific constructs (reduced expression *fos-1* with *syIs118[unc-119(+) + fos-1a::YFP-TX] I*, reduced expression *hlh-2* with *qyIs174[hlh-2p::GFP::hlh-2 + unc-119(+)]*, reduced expression *cdh-3* with *qyIs50[cdh-3>mCherry::moeABD]V* and reduced expression *unc-40* with *qyIs66[cdh-3>unc-40::gfp; myo-2::yfp] X*).

A full list of genotypes can be found in Table S2. Bottom right, sample images for pri-1i and prp-19i showing *lam-1::GFP* and *cdh-3>mCherry::moeABD*. With the red AC signal originating from two distinct locations along the body axis. Scale bar 25 µm,

Supplementary Movies

Supplementary Movie 1. Clip showcasing the device loading process. Eight separate device units are loaded consecutively. Animals are flowing in from either the left hand or right hand side of the device and pushed toward the trap channel array. Animals are maneuvered into the channel array by gently pushing and pulling on the syringe plunger. Finally once a sufficient number of animals is loaded the syringe and needle connected to a unit is removed and any animals which may have been dislodged by the removal of the needle gently pushed back into the imaging region on chip.

Supplementary Movie 2. Clip showcasing the automation workflow. Starting with an auto-focus establishing the in-focus position on the on-chip positioning markers. Followed by an overview scan identifying the location of the smallest positioning markers. Next the in-focus slice on either side of the device is identified in separate auto-focus routines, and all intermediary focus points interpolated as a linear progression. Finally, the device is imaged, acquiring two brightfield images for each group of four trap channels, covering the entire trap channel imaging region.

Supplementary Files

Supplementary File 1. CAD file for the screening device. Eight independent functional units are formed. Note the windows along the midline of the mask, which serve as alignment marker for the following mask. During alignment, the sample is viewed throught the window in this mask and the crosshair formed by the previous mask is placed at the center of the windows formed here.

Supplementary File 2. CAD file for the low height structure. This structure is fabricated first. Once exposed and hardened the structures outlined by this mask are developed and the second layer is spincoated. Note the crosshairs along the midline of the mask, which serve as alignment marker for the following mask. During alignment, make sure there is sufficient overlap between the low height and higher second structure (top and bottom). Overlap on the worm side should not be too large to avoid worms slipping from one channel into the neighboring one, but also not too small to allow sufficient liquid throughflow. Aim is an overlap of 20-35 μm**.**

Supplementary File 3. Zip file containing MATLAB script for image pre-processing (AC_FIND.m). Along with files required for YacuDecu-based image deconvolution. Parameters for AC recognition are given as variables A1, A2 and A3. Output image size is given in variable RW. The PSF for deconvolution is assumed to be preloaded and stored in using the setappdata/getappdata commands as variable 'PSF'. If no PSF file is supplied the images will not be deconvolved and may therefore not be suitable for NN-based scoring. AC_FIND expects two inputs, the file name (e.g., AH.tif) and the file path. Output files (*.png) will be stored in the same folder using the same name. Note YacuDecu does require a CUDA capable graphics card and was performed either using a NVidia RTX2080Ti or Nvidia RTX3080. With the required CUDA library preinstalled according to the manufacturer's specifications.

Supplementary File 4. MATLAB script for NN retraining and classification. Network retraining is performed using the settings given in NN_Trainer.m. Users will have to replace the path leading to the training library, with files for each category (e.g., breached, non-breached etc.) stored in subfolders with the corresponding name. NN-based classification is applied using NN_Classificaition.m. Users will have to replace the path pointing to the NN (available from https://doi.org/10.5281/zenodo.18174964, along with the used training data and raw sample images). Note NN training does require a CUDA capable graphics card and was performed either

using a NVidia RTX2080Ti or Nvidia RTX3080. With the required CUDA library preinstalled according to the manufacturer's specifications.

Table S1. List of all genes assessed in this study including gene name, sequence, previously available information on their effect on BM breaching with relevant references as well as a column indicating if a clone was imaged and its penetrance of *not-breached* in the P0 or F1. If a clone was imaged in the presented study it is indicated as [+], if it could not be imaged at all due to severe developmental delay or lethality [-]. Penetrance for all clones that could not be imaged in one or both generations, or could be imaged but were found not to affect BM breaching (i.e., achieved a low network score and were therefore not further assessed) are labeled as [-]. All newly identified clones affecting BM breaching are highlighted **bold**.

Table S2. List of all strains used in this study.

Extended Materials and Methods

SU8 Master Fabrication

Initial master molds were made from silicon wafers (Si-Wafer 4P0/>1/525±25/SSP/TTV<10, Siegert Wafer, Germany) patterned with SU8 photoresist, following standard protocols (12). Briefly, in a first step wafers were cleaned with an air plasma (Diener electronic) followed by spin-coating (WS-650-23B, Laurell) the low height layer, separating inlet from outlet area, using a low viscosity SU8 (GM1050, Gersteltec). The height of this layer was chosen to be 5 µm, high enough for liquid to pass but low enough for worms to be blocked from entering. This layer was baked and patterned using the first photomask, followed by post-baking and development. In a second step, the higher SU8 layer (20 µm) was created (GM1060, Gersteltec) baked and exposed through a second photomask, generating the space in which animals are held. Features created by the first and second mask were aligned using a mask aligner (UV KUB3, Kloe). Following exposure, wafers were post-baked and developed resulting in the final master mold. Prior to use the wafer was baked at 200°C for 10 minutes, resulting in a smooth surface and higher long-term stability of the features. The hard-baked master mold was then treated with chlorotrimethyl silane vapor (Sigma) to passivate the surface.

Step-by-step protocol

1. Clean wafers using air plasma.
2. Spincoat first SU8 layer, followed by soft-bake at 95°C.
3. Expose first layer, followed by post-bake at 95°C and development.
4. Repeat plasma cleaning after wafer is developed and dry.
5. Spincoat second SU8 layer followed by soft bake at 95°C (if necessary first bake at 65°C).
6. Expose second layer after carefully aligning the second mask to the existing features using alignment markers on either side of the wafer.
7. Post-bake and develop the wafer, followed by hard-bake at 200°C.
8. Treat wafer with chlorotrimethyl silane for at least 2 hours (NOTE 1: The silane is toxic and releases corrosive vapors. This step therefore needs to be performed in a fume hood. NOTE 2: The silane used in this step is sensitive to water, with hydrolysis resulting in the formation of hexamethyldisiloxane (2 $Me_3SiCl + H_2O \rightarrow Me_3Si\text{-}O\text{-}SiMe_3$ + 2 HCl], which does not covalently bind to the wafer surface. The silane therefore needs to be stored under

inert atmosphere. Silane bottles are typically capped with a septum. The silane should be taken from the bottle using a syringe and needle and the bottle appropriately purged with nitrogen. NOTE 3: Silanization should be repeated every few casts to ensure easy PDMS removal).

Silicone Mold Fabrication

An approximately 5 mm thick layer of polydimethylsiloxane (PDMS) elastomer (Elastosil RT601 A/B, ratio 10:1, Ameba AG) was cast on the master mold and cured at room temperature (RT). After approximately 24 hours, the PDMS was carefully removed from the wafer and cut to size. Following initial PDMS casting, the PDMS molds were manually aligned on the elevated surface of the aluminium casting dish. Once properly aligned the mold was closed and filled with freshly mixed and degassed PDMS. After curing at RT for approximately 24 hours, the cured silicone carefully removed from the aluminium mold. Silicone molds may be used without any further modification.

Step-by-step protocol

Initial PDMS mold

1. Prepare a batch of 40 g PDMS pre-polymer (Elastosil RT601 part A) and thoroughly mix it with the 4 g of crosslinker (ratio 10:1, Elastosil RT601 part B).
2. Place the wafer in an aluminium foil dish and cast the PDMS onto it. Degas the wafer for about 10 minutes and after degassing break all remaining bubbles using a pipette.
3. Cure for 24 hours at RT.
4. Remove the PDMS and cut it to size using a scalpel or razor blade. The PDMS should be cut a few millimeters larger than the original structure to ensure a flat surface.

PDMS mold alignment and Silicone Casting

1. Place the cured PDMS mold onto the elevated top surface of the aluminium mold
2. Carefully align the PDMS mold to the aluminium mold.

1. Attach sidewalls to the aluminium mold using screws.
2. Prepare a batch of PDMS sufficient to fill the entire mold cavity.
3. Degas PDMS and fill the cavity without introducing air bubbles and place the lid onto the mold

4. Leave PDMS to cure for approximately 24 hours at RT.
5. Remove the aluminium lid and inspect for larger cavities. If necessary, fill the cavities with freshly prepared PDMS and leave to cure for another 24 hours at RT. If no large cavities are present proceed.
6. Remove the sidewalls and lid, and gently remove the mold from the aluminium mold.

Polyurethane Mold Fabrication

The silicone mold was placed under vacuum for approximately 1-2 hours, removing air from the PDMS material. A batch of polyurethane (PU) was mixed (09202-001-000071, Smooth-Cast ONYX/1 SLOW, ratio 1:1 by volume, Kaupo AG) and cast into the silicone mold. The PU was left to cure for approximately 24 hours at RT. Once cured, the hardened PU was gently removed from the silicone mold, the backside flattened and mounting holes drilled into the material.

Step-by-step protocol

1. Leave the PDMS mold to degas for approximately 1-2 hours.
2. Prepare a batch of PU sufficient to fill the mold cavity (09202-001-000071, Smooth-Cast ONYX/1 SLOW, ratio 1:1 by volume, Kaupo AG) and thoroughly mix both components. NOTE: Smooth-Cast ONYX/1 SLOW is denoted as chronically toxic and harmful. Wear appropriated personal protection equipment (gloves, lab coat and goggles) and work in a well-ventilated area. Clean all equipment used and all surfaces. Wash hands.
3. Remove the silicone mold from vacuum and pour the freshly mixed PU into the mold cavity.
4. Leave PU to cure for approximately 24 hours at RT.
5. Once cured gently remove the hardened PU from the mold.
6. If needed clean all edges using a scalpel or blade. Remove any defect that may prevent proper contact between the PU surface and aluminium spacers used in subsequent steps.
7. Flatten the backside and drill mounting holes in the PU mold.

PDMS Device Fabrication

Complete molds were assembled from one patterned PU mold, an aluminium spacer and a lid, clamped together using screws, and the resulting cavity filled with freshly mixed and degassed

PDMS (Elastosil RT601A/B, Ameba). The PDMS was then left to cure for approximately 24 hours at RT. After curing the PDMS now attached to the support frame was removed, access holes punched for each device (G15 puncher, Syneo) and finally bonded to cover glass (#92101400660, Glaswarenfabrik Karl Hecht) using an air plasma (Zepto, Diener electronic) (Fig.S1M).

Step-by-step protocol

PDMS device fabrication

1. Begin by preparing all device molds. Each mold is assembled form a patterned PU mold, an aluminium spacer and a lid, clamped together with screws. Ensure all mold pieces are clean, and no gap remains between mold components with all 3 pieces well aligned to one another.
2. Prepare a batch of PDMS sufficient to fill the mold cavity (Elastosil RT601A/B). Thoroughly mix the PDMS and place in a vacuum desiccator to remove all air from the material.
3. Once the PDMS is sufficiently degassed, remove it from vacuum.
4. Remove the plunger from a suitably large syringe and place a blank tip cap on the syringe.
5. Transfer the PDMS into the syringe and place it under vacuum.
6. Once the PDMS is sufficiently degassed, remove it from vacuum.
7. While ensuring the tip cap remains on the syringe carefully put the plunger back into the syringe, invert the syringe and remove the tip cap.
8. Fill the mold cavity with PDMS.
7. Cure for approximately 24 hours at RT.
9. Remove all screws and carefully remove the lid and patterned PU mold.
10. Bond the PDMS to a cover glass using air plasma and place bonded device in the oven at 70°C for a few hours.

RNAi Plate Preparation

Prior to each experiment 1 mL of LB medium supplemented with ampicillin (0.1 mg/mL final concentration) were inoculated with bacteria from the stock LB plates and grown at 37°C overnight. Following this initial growth period additional 1 mL of LB medium supplemented with IPTG (1mM final concentration) and ampicillin (0.1 mg/mL) was added to the overnight culture

and bacteria were grown for another 3 hours to induce dsRNA synthesis (30). After this second growth phase, bacteria were harvested and seeded on RNAi plates produced by adding IPTG (1mM final concentration) and ampicillin (0.1 mg/mL final concentration) to the standard NGM plate mixture. The bacterial culture was then concentrated to approximately 1/5th of the initial volume and each NGM plate seeded with 0.3 mL of concentrate. RNAi bacteria were allowed to grow on plate, at room temperature for at least 24 hours before seeding worms.

Worm Preparation

Worms were bleached from mixed plates, with embryos left to hatch overnight in buffer (M9 or S-Basal). Once hatched worms were filtered through a 10 μm cell strainer (#43-10010-50, pluriSelect) to remove all unhatched eggs, as well as debris from the plate or leftover corpses. The L1 larvae were centrifuged at 1300 rcf (in 15mL Falcone tubes) to remove supernatant and washed with 5 mL of clean buffer. Centrifugation was then repeated, supernatant removed and worms transferred to a plate seeded with RNAi clones. Once the worms reached the desired age (approx. 36 hours), they were washed off the plate using M9 buffer and left to sediment. The supernatant was then removed and worms washed three times using fresh buffer. Once washed worms were left to sediment, with as much of the supernatant as possible being removed and a small amount of buffer containing 1wt% of Pluronic F127 (#P2443-250G, Sigma Aldrich) added.

Step-by-step protocol

1. Starting from plates with many gravid animals.
2. Wash animals of plate using M9/S-Basal buffer.
3. Add 5% NaClO and 5M NaOH to the worm suspension (200uL/100uL for every 1mL of worm suspension).
4. Gently shake bleaching mix until animals begin to break apart.
5. Centrifuge bleaching mix at 1300 rcf for 1 min.
6. Remove supernatant and add an equal amount of fresh buffer.
7. Again centrifuge at 1300 rcf for 1 min.
8. Remove the supernatant and transfer the pelleted worms to a 15mL tube with 5mL of fresh buffer.
9. Shake overnight at 20°C.

10. Filter the worm suspension through a 10μm cell strainer.
11. Centrifuge worm suspension at 1300 rcf for 1 min.
12. Remove supernatant and resuspend worms in an equal amount of buffer.
13. Centrifuge worm mixture again at 1300 rcf for 1 min.
14. Remove supernatant and transfer worms to NGM plates seeded with RNAi bacteria.
15. Once worms reach desired age, wash them of the plate using fresh M9/S-Basal.
16. Leave worms to sediment by gravity or pellet by centrifugation at 750 rcf.
17. Remove supernatant and add an equal amount of fresh buffer.
18. Repeat sedimentation of centrifugation step.
19. Remove supernatant and add an equal amount of fresh buffer.
20. Repeat sedimentation of centrifugation step for a final time.
21. Remove most of the supernatant.
22. Add a small amount of M9/SBasal containing 1wt% Pluronic F127, for a final concentration of 0.1-0.5%. (NOTE: Pluronic F127 is nontoxic and only serves as lubricant during worm loading, the precise concentration is therefore not crucial.)
23. Leave worms in the tube until worm loading.

Worm Loading

Refer to Figure 2. Begin by preparing a 15-20cm long piece of 1/16" tubing (#0642002, Tygon tubing 1/16'', Fisher Scientific) connected to a 1mL syringe, filled with M9/SBasal buffer containing 1% Pluronic F127, via a blunt needle (#300-35-970, Distrelec). Gently suck worms from the tube into the 1/16" tubing, care must be taken not to aspirate worms into the syringe. Once a sufficient amount of worms is gathered, the tubing is directly stuck into the device inlet and worms are loaded into the device channels by gently applying pressure to the syringe plunger, filling as many channels as possible. Care must be taken not to apply excessive pressure, pushing worms past the low height region into the outlet section of the device.

Step-by-step protocol

1. Start by filling a syringe with M9/SBasal buffer containing 1wt% Pluronic F127.
2. Attach a 23G needle and a long piece of 1/16" tubing to the syringe.
3. Fill the tubing with buffer from the syringe.

4. Aspirate worms from the tube into the tubing. Note do not pull the worms into the syringe and make sure no air bubbles remain in the tubing/syringe. If necessary, resuspend worms in the tube by flushing in a small amount of buffer from the syringe or tapping the tube.
5. Push the tubing into the device inlet.
6. Begin loading by gently applying pressure to the syringe buffer and monitoring device loading on a dissection scope.

Autofocus and Automatic Positioning

For autofocus and position we recommend a camera capable of acquiring more than 30 frames per second, and Z-motorization via a piezo objective drive or piezo stage, ensuring that image acquisition can occur at high rates. Slower cameras (e.g., an EMCCD) or Z-motorization through the microscope itself are possible but may substantially limit system performance. In addition, the system relies on a fast-moving XY stage (>50 mm/s), which at the same time offers high accuracy (better than 1 µm).

Autofocus and positioning are performed on a set of features fabricated along each functional unit on a device. Positioning is performed on sets of circular pillars, with 3 large and 12 medium pillars distributed on three sides of the central 4 small pillars. Pillars are positioned such that the system can reliably find its way toward the center of the 4 smallest pillars which is at a known distance from the first imaging position, after which positioning is done on a fixed, known device geometry. Focusing is likewise performed on the circular pillars as well as a number of diamond shaped features distributed around the positioning features.

Autofocus is established by acquiring a series of brightfield images, for each image in the sequence the gradient in X and Y is calculated using the Sobel method. Using these gradients, the focus measure (FM) is calculated as the standard deviation of the image gradient magnitude:

$$\mathrm{FM} = \frac{\sum_{i}\sum_{j}\sqrt{G_{X(i,j)}^{2} + G_{Y(i,j)}^{2}}}{i * j}$$

Where G_X is the gradient in X, G_Y is the gradient in Y and i and j are the number of columns and rows in each image. FM is of arbitrary magnitude with FM higher for images with higher contrast. Focus features yield highest contrast at the transition of PDMS to coverglass, the system therefore reliably focuses on the bottom of the device features.

Automatic positioning is performed by detecting the circular positioning features in brightfield images acquired at the in-focus position. Circles are detected using a circular Hough Transform, preferentially identifying the smallest circles, moving the system to the center of these four pillars. If the smallest pillars cannot be detected in the first acquired image a 3x3-tile image is acquired, covering a larger device area and detection of the smallest circles is attempted in this image. If detected the system moves to the center position, if not, detection of the medium size pillars is attempted and the system is moved to their center position. At this point acquisition of the 3x3-tile image is repeated and the smallest pillars detected moving to the final position. If the medium pillars also cannot be detected in the 3x3-tile image the largest pillars are detected, moving to their position at which point a second 3x3-tile image is acquired detecting the medium pillars followed by another 3x3-tile image, finally detecting the small pillars and moving to the center position. If the largest pillars could also not be detected, positioning fails and the system will prompt the user to manually move to the starting position and focus, at which point image acquisition can be continued.

In the following you will find a pseudocode outlining the steps above, partially adapted from MATLAB syntax. The specific way in which images are acquired will depend on the used microscope software.

Acquire a BF image at the current location, ensure flat illumination and consistent brightness.

IMG = SNAP IMAGE

Identify all circles ranging 60–80 pixels in radius, e.g., using imfindcircles a circular Hough transform.

[R1,C1] = imfindcircles(IMG,[60 80],'Sensitivity',0.925,'EdgeThreshold',0.1)

C1 and R1 outline the determined circle center and radius respectively.

Calculate the average distance between circles (m) and displacement to center (dX, dY).

CMX = mean(C1(:,1)); dx = C1(:,1) - CMX;

CMY = mean(C1(:,2)); dy = C1(:,2) - CMY;

m = mean(sqrt(dx.^2 + dy.^2));

If no circles are detected cquire a 3x3 tile image

if isempty(C1)

IMG = SNAP 3x3 IMAGE

Attempt to find smallest circles in the tiled image.

[R1,C1] = imfindcircles(IMG,[60 80],'Sensitivity',0.925,'EdgeThreshold',0.1)

If non found attempt to find medium circles in the tiled image.

[R2,C2] = imfindcircles(IMG,[80 105],'Sensitivity',0.90,'EdgeThreshold',0.1)

If non found attempt to find largest circles in the tiled image.

[R3,C3] = imfindcircles(IMG,[120 140],'Sensitivity',0.90,'EdgeThreshold',0.1)

If any of the above is successful move to the center of all identified circles. Converting pixel values to micrometer using the systems magnification (MAG) and pixel size (PXS).

e.g. **dX = round(mean(C2(:,1)))-3072; dY = round(mean(C2(:,2)))-3072;**

SetRelativeXYPosition(dX/MAG*PXS,dY/MAG*PXS)

and repeat until 4 small circles are detected

if length(C1) == 4

Again, calculate displacement from center.

dX = round(mean(C1(:,1)))-1024; dY = round(mean(C1(:,2)))-1024;

Move from current position to center of image.

SetRelativeXYPosition(dX/MAG*PXS,dY/MAG*PXS)

Move from center to first imaging location at 40x in the center of the trap channel. Specific distances depend on magnification, ROI and device type used.

Set Relative XY Position (0,420);

ACQUIRE STACK

Acquire images and move to next position

Set Relative XY Position (0,300);

ACQUIRE STACK

Image Processing and Transfer Learning

Image pre-processing is performed on multicolor (GFP, mCherry and DIC) TIFF-stacks with 3x41 slices. Initially only the red portion is loaded into MATLAB and maximum z-projected. At this point a set threshold (0.005, MATLAB *imbinarize*) is applied to the projection, and the image cleared of any objects, too small, too large or outside of a user specified intensity range (1000-50000 pixels and a required maximum brightness of 500 counts). This process reliably identifies the position of the AC/VPCs outlined in red. Following focusing and identification of the region

of interest (ROI), the complete stack within a 376x376 pixel window around the identified location is loaded into MATLAB and deconvolved using the YacuDecu (35) implementation of CUDA-based Richardson-Lucy deconvolution (50 iterations, carried out for GFP and mCherry, on an Nvidia RTX3080), using a computer-generated point spread function (PSFGenerator, ImageJ, (36)). Once deconvolved the red intensity standard deviation (SD) is calculated for each frame, with the frame showing the highest SD assumed to be in focus. Finally, an auto-contrast is applied to all in-focuse images by saturating the bottom and the top 1% of all pixel values (MATLAB *imadjust*). Auto-contrast was applied to ensure consistent lighting across all images, effectively eliminating brightness of the fluorescent markers as a variable during NN training/classification. Images are then assembled into an RGB image and saved as .png files. PNGs were selected as they are lightweight, introduce little-to-no compression artefacts and can easily be inspected for manual annotation.

Image pre-processing was carried out the same for images used in transfer learning and screening images classified by the NN. At this stage all training images were manually classified.

Transfer learning was carried out as described here (37). Initially sorted images were imported into a MATLAB imagedatastore, designating the four classes which need to be differentiated. The created imagedatastore was split at a ratio of 70:30 (training:validation) and random permutations (rotation, translation, mirroring, scaling and shearing) were applied to the training dataset. These permutations were periodically reapplied to further randomize the dataset. Next a pretrained NN (Inception V3) was imported and the existing output layers removed (layer 313 and 315) and replaced with a new learnable and class layer. Additionally, the first 17 layers were frozen as these are responsible for basic image pre-processing, and likely don't affect network decision making. Finally, training parameters were set (see Supplementary File 4, NN_Trainer.m) and the network training executed using the MATLAB *trainNetwork* utility, and the retrained NN stored. Training parameters (*primarily learning rate, learning rate drop and period, and regularization*) were iteratively adjusted such that the NN yielded high accuracy and low loss when scoring the validation portion of the dataset.

For image classification the retrained network was loaded into MATLAB. Images to be classified were loaded from file. Images were then passed on to the NN and the resulting scores assigned to each of the four output classes recorded. Alternatives NN classification was implemented as part of the pre-processing, directly passing the RGB image to the classifier, prior to storage as .png.

All network assigned scores were collected and RNAi clones sorted according to the number of presumably non-breached animals (network scores) and all clones of interest validated manually.

How to adapt the outlined processing strategy to newly generated image data?

Preprocessing

Requires MATLAB and the Image Processing Toolbox, as well as installation of the CUDA SDK (select a version compatible with your MATLAB installation and your GPU) and MinGW-w64 compiler installed in MATLAB which can be obtained from the MATLAB App store APPS tab > Get More Apps .

Assuming that newly generated multi-color image data follows the same channel order employed in the presented work (GFP, mCherry, and DIC) and images are stored as .tif stacks (with alternating colors and Z slices, Z1C1, Z1C2, Z1C3, Z2C1, Z2C2, Z2C3, etc.), limited changes will need to be made to the preprocessing pipeline. Preprocessing requires a manually set value for binarization threshold (currently A2 = 0.005) and minimum object brightness (currently A3 = 1000) to account for possible changes in image brightness compared to the ones acquired on our system.

Furthermore, users may need to adjust the minimum object size (currently A1 = 500 pixels) of a camera with different pixel size (currently 6.5 um) or magnification (currently 40x) is used. However, it should be noted the pre-processing code expects images acquired at a specific size and animals in a linear format, with the image width > image height. As such ROI position is only determined along the image width and the full height (376 pixels) used for further processing. If animals in a non-linear format (e.g., on an agar pad) are imaged the code will need to be adjusted to locate the ROI along width and height.

Finally, users will need to provide a suitable point spread function (PSF) for deconvolution, imported into MATLAB and stored appropriately. This can be accomplished using the pseudocode provided below:

```
PTH = 'PSF location .tif'; n = 1;
for ZZ = 1:NUMBER OF ZSLICES % here 41
    for CC = 1:NUMBER OF COLORS % here 2 (GFP and mCherry, not DIC)
        PSF(:,:,ZZ,CC) = imread(PTH,n); n = n + 1;
```

end

end

setappdata(0,'PSF',PSF); % stores the variable in MATLAB.

Once suitable parameters have been found preprocessing should function as described, generating images of a fixed output format (299x299x3 RGB images in .png format, with a fixed contrast range saturating the top and bottom 1% of the image histogram) and newly generated may be possible to analyze right away with the retrained NN provided here (**https://doi.org/10.5281/zenodo.18174964**).

We provide the function AC_FIND.m, which can be called from the MATLAB command window or as part of a for loop moving through a list of images. For each image AC_FIND needs to be called with the images file name and path to the folder in which it is located. Such a file list can be generated using the **imageDatastore** function in MATLAB.

NN retraining

Requires MATLAB, the Deep Learning Toolbox and the desired CNN in our case Inception V3, which can be obtained from the MATLAB App store APPS tab > Get More Apps, and ideally a compatible GPU.

If required retraining a convolutional NN (CNN) begins with manual annotation of the training data and sorting of the generated .png images into separate subfolders, contained in one parent folder. In the file NN_Trainer.m replace the value 'INSERT FOLDER PATH' in line 3 with the parent folder path and run the code.

imds = imageDatastore('INSERT FOLDER PATH','IncludeSubfolders',1,'LabelSource','foldername');

The code begins by splitting the training data into 70% training and 30% validation, with training category names/labels taken from the subfolder names generated above. Next layer 313 (the last fully connected layer) and 315 (the output layer) are removed and replaced with the same type of layer but without any preexisting training, such that new output categories can be trained. Note: If a different type of CNN is used different layer numbers will need to be removed.

Next the initial 17 layers are frozen, i.e., their training weights fixed. The initial layers largely classify basic image properties and do not need to be retrained for a new application.

layers = lgraph.Layers;

```
connections = lgraph.Connections;
layers(1:17) = freezeWeights(layers(1:17));
```

Next images are augmented, i.e., treated with a random set of operations such as mirroring, rotation, scaling or shearing, artificially increasing the training dataspace, yielding a more robust result.

```
aug = imageDataAugmenter('RandXReflection',1, ...
    'RandYReflection',1, ...
    'RandRotation',[-180 180], ...
    'RandScale',[0.8 1.0], ...
    'RandXShear',[-10 10], ...
    'RandYShear',[-10 10], ...
    'RandXTranslation',[-25 25], ...
    'RandYTranslation',[-25 25]);
```

Finally, the training is started with a given set of parameters, empirically changing the values with the aim of yielding a newly trained NN with high accuracy and low loss.

```
miniBatchSize = 64;
valFrequency = floor(numel(auimdsTrain.Files)/miniBatchSize);
options = trainingOptions('sgdm', ...
    'MiniBatchSize',miniBatchSize, ...
    'MaxEpochs',100, ...
    'InitialLearnRate', 7.5e-3, ...
    'LearnRateSchedule','piecewise', ...
    'LearnRateDropFactor',0.1, ...
    'LearnRateDropPeriod', 10, ...
    'Shuffle','every-epoch', ...
    'ValidationData',auimdsValidation, ...
    'ValidationFrequency',valFrequency, ...
    'L2Regularization',0.05, ...
    'Verbose',true, ...
    'VerboseFrequency', 100, ...
    'Plots','training-progress');
```

sgdm: Stochastic gradient descent with momentum (SGDM). Note other solvers are available and have different parameters.

MaxEpochs: Maximum number of epochs (full passes of the data) to use for training, specified as a positive integer.

MiniBatchSize: Size of the mini-batch to use for each training iteration, specified as a positive integer. A mini-batch is a subset of the training set that is used to evaluate the gradient of the loss function and update the weights.

InitialLearnRate: Initial learning rate used for training, specified as a positive scalar. If the learning rate is too low, then training can take a long time. If the learning rate is too high, then training might reach a suboptimal result or diverge.

LearnRateSchedule: Option for dropping the learning rate during training, specified as one of these values:

- **none**: Keep learning rate constant throughout training.
- **piecewise**: Update the learning rate periodically using a drop factor.

LearnRateDropPeriod: Number of epochs after which the learning rate is dropped.

LearnRateDropFactor: Factor for dropping the learning rate, specified as a scalar from 0 to 1.

Momentum: Contribution of the previous iteration to the current iteration, specified as a scalar from 0 to 1.

MaxIterations: Maximum number of iterations to use for training, specified as a positive integer.

Shuffle: Option for data shuffling, specified as one of these values:

- **once:** Shuffle the training and validation data once before training.
- **never:** Do not shuffle the data.
- **every-epoch:** Shuffle the training data before each training epoch, and shuffle the validation data before each neural network validation.

L2Regularization: Factor for L2 regularization (weight decay), specified as a nonnegative scalar. Adding a regularization term for the weights to the loss function is one way to reduce overfitting.

Adapted from MathWorks documentation.

NN classification

Requires MATLAB, the Deep Learning Toolbox and the desired retrained NN.

Replace the path in line 53 with the one pointing to the retrained NN downloaded from Zenodo or generated using the above function. Run the code NN_Classification.m and point the system at the parent folder containing your preprocessed data. The script will first generate a list of all .png files found in up to 5 layers of subfolders and will finally present the images to the NN for classification. NN scores are then recorded and stored in a .xlsx file alongside the original data (i.e., in the related subfolder).

REFERENCES

1. K. Chung, M. M. Crane, H. Lu, Automated on-chip rapid microscopy, phenotyping and sorting of *C. elegans*. *Nat. Methods* **5**, 637–643 (2008).

2. A. San-Miguel, P. T. Kurshan, M. M. Crane, Y. Zhao, P. T. McGrath, K. Shen, H. Lu, Deep phenotyping unveils hidden traits and genetic relations in subtle mutants. *Nat. Commun.* **7**, 12990 (2016).

3. S. Mondal, E. Hegarty, C. Martin, S. K. Gökce, N. Ghorashian, A. Ben-Yakar, Large-scale microfluidics providing high-resolution and high-throughput screening of *Caenorhabditis elegans* poly-glutamine aggregation model. *Nat. Commun.* **7**, 13023 (2016).

4. S. Spiri, S. Berger, L. Mereu, A. DeMello, A. Hajnal, Reciprocal EGFR signaling in the anchor cell ensures precise inter-organ connection during *Caenorhabditis elegans* vulval morphogenesis. *Development* **149**, dev199900 (2022).

5. D. R. Sherwood, P. W. Sternberg, Anchor cell invasion into the vulval epithelium in *C. elegans*. *Dev. Cell* **5**, 21–31 (2003).

6. E. J. Hagedorn, J. W. Ziel, M. A. Morrissey, L. M. Linden, Z. Wang, Q. Chi, S. A. Johnson, D. R. Sherwood, The netrin receptor DCC focuses invadopodia-driven basement membrane transmigration in vivo. *J. Cell Biol.* **201**, 903–913 (2013).

7. D. R. Sherwood, J. A. Butler, J. M. Kramer, P. W. Sternberg, FOS-1 promotes basement-membrane removal during anchor-cell invasion in *C. elegans*. *Cell* **121**, 951–962 (2005).

8. L. C. Kelley, Q. Chi, R. Cáceres, E. Hastie, A. J. Schindler, Y. Jiang, D. Q. Matus, J. Plastino, D. R. Sherwood, Sherwood adaptive F-actin polymerization and localized ATP production drive basement membrane invasion in the absence of MMPs. *Dev. Cell* **48**, 313–328.e8 (2019).

9. Q. Yang, D. Roiz, L. Mereu, M. Daube, A. Hajnal, The invading anchor cell induces lateral membrane constriction during vulval lumen morphogenesis in *C. elegans*. *Dev. Cell* **42**, 271–285.e3 (2017).

10. D. R. Sherwood, J. Plastino, Invading, leading and navigating cells in *Caenorhabditis elegans*: Insights into cell movement in vivo. *Genetics* **208**, 53–78 (2018).

11. I. W. Kenny-Ganzert, D. R. Sherwood, The *C. elegans* anchor cell: A model to elucidate mechanisms underlying invasion through basement membrane. *Semin. Cell Dev. Biol.* **154**, 23–34 (2024).

12. Y. Xia, G. M. Whitesides, Soft lithography. *Angew. Chem. Int. Ed. Engl.* **37**, 550–575 (1998).

13. R. S. Kamath, A. G. Fraser, Y. Dong, G. Poulin, R. Durbin, M. Gotta, A. Kanapin, N. Le Bot, S. Moreno, M. Sohrmann, D. P. Welchman, P. Zipperlen, J. Ahringer, Systematic functional analysis of the *Caenorhabditis elegans* genome using RNAi. *Nature* **421**, 231–237 (2003).

14. T. Deng, P. Stempor, A. Appert, M. Daube, J. Ahringer, A. Hajnal, E. Lattmann, The *Caenorhabditis elegans* homolog of the Evi1 proto-oncogene, egl-43, coordinates G1 cell cycle arrest with pro-invasive gene expression during anchor cell invasion. *PLOS Genet.* **16**, e1008470 (2020).

15. M. K. Morf, I. Rimann, M. Alexander, P. Roy, A. Hajnal, The *Caenorhabditis elegans* homolog of the Opitz syndrome gene, madd-2/Mid1, regulates anchor cell invasion during vulval development. *Dev. Biol.* **374**, 108–114 (2013).

16. P. Davis, M. Zarowiecki, V. Arnaboldi, A. Becerra, S. Cain, J. Chan, W. J. Chen, J. Cho, E. da Veiga Beltrame, S. Diamantakis, S. Gao, D. Grigoriadis, C. A. Grove, T. W. Harris, R. Kishore, T. Le, R. Y. N. Lee, M. Luypaert, H.-M. Müller, C. Nakamura, P. Nuin, M. Paulini, M. Quinton-Tulloch, D. Raciti, F. H. Rodgers, M. Russell, G. Schindelman, A. Singh, T. Stickland, K. Van Auken, Q. Wang, G. Williams, A. J. Wright, K. Yook, M. Berriman, K. L. Howe, T. Schedl, L. Stein, P. W. Sternberg, WormBase in 2022—Data, processes, and tools for analyzing *Caenorhabditis elegans*. *Genetics* **220**, iyac003 (2022).

17. B. J. Hwang, A. D. Meruelo, P. W. Sternberg, *C. elegans* EVI1 proto-oncogene, EGL-43, is necessary for Notch-mediated cell fate specification and regulates cell invasion. *Development* **134**, 669–679 (2007).

18. I. Rimann, A. Hajnal, Regulation of anchor cell invasion and uterine cell fates by the egl-43 Evi-1 proto-oncogene in *Caenorhabditis elegans*. *Dev. Biol.* **308**, 187–195 (2017).

19. D. Q. Matus, L. L. Lohmer, L. C. Kelley, A. J. Schindler, A. Q. Kohrman, M. Barkoulas, W. Zhang, Q. Chi, D. R. Sherwood, Invasive cell fate requires G1 cell-cycle arrest and histone deacetylase-mediated changes in gene expression. *Dev. Cell* **35**, 162–174 (2015).

20. E. J. Hagedorn, H. Yashiro, J. W. Ziel, S. Ihara, Z. Wang, D. R. Sherwood, Integrin acts upstream of netrin signaling to regulate formation of the anchor cell's invasive membrane in *C. elegans*. *Dev. Cell* **17**, 187–198 (2009).

21. J. W. Ziel, E. J. Hagedorn, A. Audhya, D. R. Sherwood, UNC-6 (netrin) orients the invasive membrane of the anchor cell in *C. elegans*. *Nat. Cell Biol.* **11**, 183–189 (2009).

22. C. Szegedy, V. Vanhoucke, S. Ioffe, J. Shlens, Z. Wojna, Rethinking the inception architecture for computer vision, in *Proceedings of the IEEE Conference on Computer Vision and Pattern Recognition* (IEEE, 2016), pp. 2818–2826.

23. L. P. O'Reilly, C. J. Luke, D. H. Perlmutter, G. A. Silverman, S. C. Pak, *C. elegans* in high-throughput drug discovery. *Adv. Drug Deliv. Rev.* **69**, 247–253 (2014).

24. F. Simmer, M. Tijsterman, S. Parrish, S. P. Koushika, M. L. Nonet, A. Fire, J. Ahringer, R. H. Plasterk, Loss of the putative RNA-directed RNA polymerase RRF-3 makes *C. elegans* hypersensitive to RNAi. *Curr. Biol.* **12**, 1317–1319 (2002).

25. R. S. Kamath, M. Martinez-Campos, P. Zipperlen, A. G. Fraser, J. Ahringer, Effectiveness of specific RNA-mediated interference through ingested double-stranded RNA in *Caenorhabditis elegans*. *Genome Biol.* **2**, RESEARCH0002 (2001).

26. S. I. Grewal, The molecular basis of heterochromatin assembly and epigenetic inheritance. *Mol. Cell* **83**, 1767–1785 (2023).

27. E. Lattmann, T. Deng, M. Walser, P. Widmer, C. Rexha-Lambert, V. Prasad, O. Eichhoff, M. Daube, R. Dummer, M. P. Levesque, A. Hajnal, A DNA replication-independent function of pre-replication complex genes during cell invasion in *C. elegans*. *PLOS Biol.* **20**, e3001317 (2022).

28. S. Brenner, The genetics of *Caenorhabditis elegans*. *Genetics* **77**, 71–94 (1974).

29. C. Frøkjær-Jensen, M. W. Davis, M. Sarov, J. Taylor, S. Flibotte, M. LaBella, A. Pozniakovsky, D. G. Moerman, E. M. Jorgensen, Random and targeted transgene insertion in *Caenorhabditis elegans* using a modified Mos1 transposon. *Nat. Methods* **11**, 529–534 (2014).

30. D. Conte Jr., L. T. MacNeil, A. J. Walhout, C. C. Mello, RNA Interference in *Caenorhabditis elegans*. *Curr. Protoc. Mol. Biol.* **109**, 26.3.1–26.3.30 (2015).

31. T. Stiernagle, Maintenance of *C. elegans*. WormBook: The online review of *C. elegans* biology [Internet] (2006).

32. M. Kirouac, P. W. Sternberg. cis-Regulatory control of three cell fate-specific genes in vulval organogenesis of *Caenorhabditis elegans* and C. briggsae. *Dev. Biol.* **257**, 85–103 (2003).

33. A. Fergin, G. Boesch, N. R. Greter, S. Berger, A. Hajnal, Tissue-specific inhibition of protein sumoylation uncovers diverse SUMO functions during *C. elegans* vulval development. *PLOS Genet.* **18**, e1009978 (2022).

34. D. G. Gibson, L. Young, R. Y. Chuang, J. C. Venter, C. A. Hutchison III, H. O. Smith, Enzymatic assembly of DNA molecules up to several hundred kilobases. *Nat. Methods* **6**, 343–345 (2009).

35. B. Pepin, GPU Deconvolution Library with C and Matlab API for nVidia GPUs [online] (2025).

36. H. Kirshner, F. Aguet, D. Sage, M. Unser, 3-D PSF fitting for fluorescence microscopy: Implementation and localization application. *J. Microsc.* **249**, 13–25 (2013).

37. MATLAB Get started with transfer learning. https://mathworks.com/help/deeplearning/gs/get-started-with-transfer-learning.html.
